# Debridement, antimicrobial therapy, and implant retention (DAIR) as curative strategy for acute periprosthetic hip and knee infections: a position paper of the European Bone & Joint Infection Society (EBJIS)

**DOI:** 10.5194/jbji-10-101-2025

**Published:** 2025-04-01

**Authors:** Irene K. Sigmund, Tristan Ferry, Ricardo Sousa, Alex Soriano, Willem-Jan Metsemakers, Martin Clauss, Rihard Trebse, Marjan Wouthuyzen-Bakker

**Affiliations:** 1 Department of Orthopaedics and Traumatology, Medical University of Vienna, Vienna, Austria; 2 Infectious and Tropical Diseases Unit, Croix-Rousse Hospital, Hospices Civils de Lyon, Université Claude Bernard Lyon 1, Lyon, France; 3 Porto Bone Infection Group (GRIP), Orthopaedic Department, ULS Santo António, Porto, Portugal; 4 Hospital Clinic of Barcelona, Barcelona, Spain; 5 IDIBAPS, Barcelona, Spain; 6 CIBERNIF, CIBER in Infectious Diseases, ISCIII, Madrid, Spain; 7 Department of Trauma Surgery, University Hospitals Leuven, Leuven, Belgium; 8 Department of Development and Regeneration, KU Leuven, Leuven, Belgium; 9 Department of Orthopaedics and Trauma Surgery, Center for Musculoskeletal Infections (ZMSI), University Hospital Basel, Basel, Switzerland; 10 Orthopaedic Hospital Valdoltra, Ankaran, Slovenia; 11 Department of Medical Microbiology and Infection Prevention, University Medical Center Groningen, University of Groningen, Groningen, the Netherlands

## Abstract

It has been shown that the outcome of a DAIR (debridement, antimicrobial therapy, and implant retention) procedure depends on multiple factors (e.g. infection type, host factors, clinical presentation, condition of surrounding soft tissue, causing pathogen, surgical technique, antimicrobial therapy); therefore, adequate patient selection is key for DAIR success. In this position paper, we discuss the most relevant factors influencing the outcome and define indications, contraindications, and risk factors for a DAIR procedure based on the most robust and most recently published data. Furthermore, we discuss the surgical technique in combination with systemic antimicrobial therapy in patients undergoing a DAIR procedure.

This position paper may help reduce reinfection rates as well as the physical, psychological, and economic burden associated with periprosthetic joint infection (PJI). We believe that a reasonable outcome can be achieved with careful patient selection, a dedicated multidisciplinary team, and an appropriate surgical technique and antimicrobial therapy.

## 1 Introduction

Prosthetic joint replacement revolutionized the management of patients suffering from osteoarthritis and secondary arthritis by providing long-term pain relief, restoration of limb and joint function, and improved mobility, leading to an increased quality of life and independence. Furthermore, it is described as one of the most cost-effective healthcare procedures (Lavernia et al., 1997; Chang et al., 1996; Ethgen et al., 2004).

Due to the success of this intervention and an ageing population, the numbers have soared worldwide during the last decades and are expected to rise continuously over the next years. It is projected that in 2040 more than 1.4 million total hip arthroplasties (THAs) (estimated increase compared to the 2014 US nationwide inpatient sample numbers: 284 %) and more than 3.4 million total knee arthroplasties (TKAs) (estimated increase: 401 %) will be performed in the United States alone (Singh et al., 2019).

Although this procedure is called “operation of the century” (Learmonth et al., 2007), serious complications can occur intra- and postoperatively (Katz et al., 2021). Due to the estimated increase of primary arthroplasties in the future, the number of revision surgeries is expected to rise simultaneously. From 2014 to 2030, the incidence of revision after THA is projected to increase by between 43 % and 70 % and after TKA by between 78 % and 182 % (Schwartz et al., 2020).

Arguably the most feared complication after a total joint replacement is a periprosthetic joint infection (PJI). If not treated accurately, PJI can lead to multiple revision surgeries, prolonged antimicrobial therapies and hospitalization, substantial patient morbidity, increased mortality, impairment of function resulting in a high physical and psychological burden for the patient, and an increased logistic and economic burden for healthcare systems (Zmistowski et al., 2013; Shahi et al., 2017; Kurtz et al., 2018; Vanhegan et al., 2012; Haddad et al., 2017; Fischbacher et al., 2018; Sousa et al., 2018; Schwartz et al., 2020). Despite improvements in PJI prevention (e.g. methicillin-resistant *Staphylococcus aureus* (MRSA) screening, preoperative prophylactic antimicrobial therapy), the incidence of PJI following primary replacement ranges between 1 % and 2 % (Kurtz et al., 2010; Gundtoft et al., 2015; Huotari et al., 2015). Furthermore, this number increases to approximately 4 % after revision surgery (Kurtz et al., 2007; Ong et al., 2009). In general, the aim of PJI management (surgical and antimicrobial therapy) is to eradicate the infection, provide pain relief, minimize patient morbidity, and provide sufficient mobility for the patient.

Curative surgical treatment strategies which can provide limb salvage and preserve sufficient joint function are debridement, antimicrobial therapy, irrigation, and implant retention (DAIR); one-stage exchange; and two-stage exchange (Garvin and Hanssen, 1995). Severely immunocompromised patients (e.g. solid organ transplant recipients, drug abuse, use of chemotherapy), patients with poor soft tissue conditions or bone loss, patients with an infection caused by highly resistant pathogens, and patients without the need of a functional prosthesis (e.g. paralysis) may benefit from resection arthroplasty, arthrodesis, or amputation. Long-term suppressive antimicrobial therapy may be considered in patients with a high intraoperative risk and multiple severe comorbidities where revision arthroplasty is contraindicated, in patients without further surgical option for reconstruction and sufficient functional outcome, or in patients who refuse surgery (infection control rather than eradication).

The definitive decision depends on numerous variables including infection type, prosthesis stability, causing microorganism and its susceptibility patterns, condition of the surrounding soft tissues, extent of bone loss, the available options for successful reconstruction of the joint, host factors (e.g. comorbidities, age, fragility), and surgeon's experience. It is recommended that the definitive decision of further treatment is made by a multidisciplinary team (involving different specialists including orthopaedic surgeons, infectious disease specialists, microbiologists, radiologists, and – if necessary – plastic surgeons) on a case-by-case basis (Osmon et al., 2013; Bernaus et al., 2022).

In this position paper, we discuss the most relevant factors influencing the DAIR outcome based on the most robust and currently available literature and define indications, contraindications, and risk factors for a DAIR procedure when infection eradication is intended. Furthermore, we provide a comprehensive guide on the surgical technique and antimicrobial therapy for the management of patients undergoing a DAIR procedure.

## 2 Debridement, antimicrobial therapy, and implant retention (DAIR)

In contrast to revision surgery, DAIR is a technically less demanding procedure and may prevent an unnecessary removal of a soundly fixed prosthesis with the advantage of bone stock preservation and avoidance of intraoperative complications (e.g. fractures). Under appropriate circumstances, a successful DAIR procedure can avoid revision arthroplasty procedures and the associated anaesthetic risks. It can preserve a functioning implant and allows an earlier return to activity (Grammatopoulos et al., 2017b; Dzaja et al., 2015). Further benefits are shorter operation times and hospital stays, lower patient morbidity, better postoperative mobility (compared with revision surgery), and lower economic costs (Grammatopoulos et al., 2017a; Sousa et al., 2018; Puhto et al., 2019; Sherrell et al., 2011).

**Table 1:** Literature regarding reinfection rate after a DAIR (debridement, antimicrobial therapy, and implant retention) procedure. EA – early acute infection), AH – acute haematogenous infection. NA – not available.

Literature	DAIR	Study design	Type of	Location	Failure
	( n )		infection		rate
Byren et al. (2009)	112	Retrospective 1998–2003	All PJIs	Hip ( n=52 ), knee ( n=51 ), other ( n=9 , ankle, shoulder, elbow)	18 %
Cobo et al. (2011)	117	Prospective 2004–2006	EA	Hip ( n=84 ), knee ( n=52 ), shoulder ( n=2 )	43 %
Kuiper et al. (2013)	91	Retrospective 2004–2009	NA	Hip ( n=62 ), knee ( n=29 )	34 %
Lora-Tamayo et al. (2013)	345	Retrospective 2003–2010	EA, LA	Hip ( n=146 ), knee ( n=195 ), other ( n=4 )	45 %
Triantafyllopoulos et al. (2015)	78	Retrospective 2000–2013	EA, LA	Knee	45 %
Zmistowski et al. (2016)	153	Retrospective 2000–2013	NA	Hip ( n=60 ), knee ( n=93 )	48 %
Grammatopoulos et al. (2017a)	82	Retrospective 1997–2013	All PJIs	Hip	32 %
Fink et al. (2017)	67	Retrospective 2004–2013	EA, LA	Hip ( n=23 ), knee ( n=44 )	28 %
Swenson et al. (2018)	72	Retrospective 2004–2013	EA, LA	Hip, knee	26 %
Jacobs et al. (2019)	91	Retrospective 2012–2014	EA	Hip ( n=51 ), knee ( n=40 )	15 %
Ottesen et al. (2019)	58	Retrospective 2008–2013	EA	Knee	16 %
Wouthuyzen-Bakker et al. (2019)	340	Retrospective 2005–2015	LA	Hip, knee	45 %
Clauss et al. (2020)	58	Retrospective 1992–2016	EA, LA	Hip	7 %
Becker et al. (2020)	79	Retrospective 2011–2016	EA	Hip ( n=59 ), knee ( n=21 )	32 %
Shohat et al. (2020)	1174	Retrospective 2005–2017	EA, LA	Hip ( n=565 ), knee ( n=609 )	35 %
Svensson et al. (2020)	575	Retrospective 2009–2016	NA	Hip	34 %
Tirumala et al. (2021)	149	Retrospective NA	EA, LA	Hip ( n=59 ), knee ( n=90 )	17 %
Toh et al. (2021)	106	Retrospective 2003–2018	EA	Knee	30 %
Zhu et al. (2021)	230	Retrospective 2001–2015	All PJIs	Knee	46 %
Nurmohamed et al. (2021)	67	Retrospective 2009–2017	EA, LA	Hip ( n=41 ), knee ( n=26 )	34 %
Perez et al. (2022)	63	Retrospective	EA, LA	Hip ( n=19 ), knee ( n=44 )	46 %
Tarity et al. (2022)	40	Retrospective 2013–2018	EA, LA	Hip ( n=12 ), knee ( n=28 )	35 %
Chang et al. (2022)	101	Retrospective 2005–2019	EA, LA	Knee	23 %
Rahardja et al. (2023)	189	Prospective 2014–2017	All PJIs	Knee	55 %
Sigmund et al. (2024)	176	Retrospective 2010–2020	All PJIs	Hip ( n=80 ), knee ( n=106 )	38 %

Although DAIR is a less invasive procedure and shows multiple advantages, patients should be selected carefully by the treating multidisciplinary team before undergoing this surgical option. It has been shown that, depending on patient selection, a high percentage of PJI patients treated with DAIR failed (Table 1). Hence, it is essential to identify the subset of patients who will benefit from this procedure. Therefore, different authors and societies defined indications for DAIR: in 2004, Zimmerli et al. (2004) published a review describing the management of PJI. According to their algorithm, DAIR is a reasonable option for patients with an early or late acute infection, if the duration of clinical signs and symptoms is <3 weeks, the implant is stable, the soft tissue is in good condition, and an antimicrobial agent with activity against in biofilm-embedded microorganisms is available (Zimmerli et al., 2004). In 2013, the Infectious Diseases Society of America (IDSA) published their guidelines for the diagnosis and management of PJI (Osmon et al., 2013). Based on their algorithm, a DAIR should be considered in PJI patients with a well-fixed prosthesis, without a sinus tract, who are within approximately 30 d of prosthesis implantation (early acute), or <3 weeks of symptom onset (late acute) (Osmon et al., 2013). In addition, the causing microorganism should be susceptible to oral antimicrobial agents (defined as an agent that can be used for long-term treatment or an agent which is active against in biofilm-embedded microorganisms). The most recent recommendations regarding DAIR indications were published in 2018 after the International Consensus Meeting (ICM) in Philadelphia (ICM, 2018). Here, a DAIR procedure is indicated in patients with an early ( <30 d after index arthroplasty) or late acute infection as long as the onset of symptoms is <4 weeks (favourable <7 d), implants are well fixed, no sinus tract exists, and the isolated infecting organism is sensitive to an antimicrobial agent. This was agreed by 94 % of delegates (ICM, 2018). In a review by Tsang et al. (2017), a decrease of the reinfection rate after 2004 was reported, and they suggested that there might have been a learning effect after the introduction of the first PJI management guideline in 2004 (Zimmerli et al., 2004) and the subsequent identification of risk factors associated with DAIR failure in the literature (Tsang et al., 2017). Similar results were reported by Bedair et al. (2020), with increasing success rates after 2004. However, the level of evidence in most guidelines is only reported as “limited” or “moderate”. In addition, the failure rate after a DAIR procedure still varies widely and ranged in the more recently published articles between 7 % and 55 % (Byren et al., 2009; Cobo et al., 2011; Kuiper et al., 2013; Lora-Tamayo et al., 2013; Triantafyllopoulos et al., 2015; Zmistowski et al., 2016; Grammatopoulos et al., 2017a; Fink et al., 2017; Swenson et al., 2018; Jacobs et al., 2019; Ottesen et al., 2019; Wouthuyzen-Bakker et al., 2019; Clauss et al., 2020; Becker et al., 2020; Shohat et al., 2020; Svensson et al., 2020; Tirumala et al., 2021; Toh et al., 2021; Zhu et al., 2021; Nurmohamed et al., 2021; Perez et al., 2022; Tarity et al., 2022; Chang et al., 2022; Rahardja et al., 2023; Sigmund et al., 2024) (Table 1). Possible explanations for these diverging outcomes are the differences among the studies regarding study designs, diagnostic criteria, indications for DAIR (e.g. type of infection, definition of acute infection), length of follow-up periods, definitions of “success”, duration and regimens of antimicrobial therapies, patient comorbidities, causing microorganisms, surgical technique, and number of performed DAIRs.

An enhanced understanding of the characteristics of DAIR failure may allow a more targeted approach and may lead to an improved outcome and increased infection eradication rate.

In the last decade, various host- and implant-related factors were described to be associated with a higher risk of DAIR failure, for example the timing of debridement/duration of symptoms (Tsang et al., 2017; Grammatopoulos et al., 2017a; Triantafyllopoulos et al., 2015), type of infection (early vs. late acute) (Wouthuyzen-Bakker et al., 2020b; Fink et al., 2017), presence of bacteraemia (Lora-Tamayo et al., 2013, 2017; Wouthuyzen-Bakker et al., 2019; Löwik et al., 2018), host status (Azzam et al., 2010; Bernaus et al., 2022), causing microorganism (Byren et al., 2009; Löwik et al., 2018; Azzam et al., 2010; Katakam et al., 2020a), previous revision surgeries (Byren et al., 2009; Wouthuyzen-Bakker et al., 2019), exchange of mobile parts (Shohat et al., 2020; Zhu et al., 2021), and antimicrobial treatment strategy (Zimmerli et al., 1998; Holmberg et al., 2015; Rodríguez-Pardo et al., 2014). However, the described factors are not always confirmed by others and will be discussed in more detail below.

## 3 Indications for a DAIR procedure

In general, a DAIR (as curative option) is indicated in patients with an acute PJI (early as well as late acute) and a soundly fixed prosthesis. However, different factors were associated with a higher risk of DAIR failure. In this section, the indications and contraindications for a DAIR procedure as well as risk factors for failure are discussed based on more recently published data. In addition, upcoming controversies and debates about various risk factors are addressed.

### 3.1 Type of infection

Due to higher failure rates, chronic infections are considered an absolute contraindication to perform a DAIR, as concluded at the ICM 2018 (Argenson et al., 2019). Indeed, Davis et al. (2022) observed in their prospective observational study of 569 PJIs (acute: n=427 , chronic: n=142 ) a statistically significant higher failure rate in chronic infections (56 %; defined as >30 d after the original arthroplasty and/or >30 d of symptoms) compared to acute PJIs (41 %, p=0.003 ). Similar results were demonstrated by Zhu et al. (2021) in their retrospective study of 230 infected total knee arthroplasties (acute ( <90 d): n=107 , failure rate: 34 %; chronic ( >90 d): n=123 , failure rate: 57 %; p=0.0004 ) and Rahardja et al. (2023) in their prospective study of 189 PJIs following primary total knee arthroplasty (acute ( <90 d): n=60 , failure rate: 37 %; chronic ( >90 d): n=129 , failure rate: 64 %; p=0.001 ; OR 3.08, 95 % CI: 1.41–6.72, p=0.05 ).

The main impetus for dividing PJIs into “acute” and “chronic” infections is the ability of microorganisms to form biofilms on implants or necrotic bone fragments. Fully developed mature biofilm matrixes protect bacteria from the host immune system and significantly minimize the efficacy of antibiotics by different mechanisms. Bacteria in the deep biofilm stratum significantly reduce their metabolism and become tolerant to antibiotics (“small colony variants”). In addition, the matrix protects a small subpopulation ( <0.1 %) of spontaneously generated dormant cells present in any bacterial colony highly tolerant to almost all antibiotics (so-called “persister cells”) that normally are easily eliminated by phagocytes (Hamad et al., 2022). Once maturation is achieved, it is assumed that biofilm bacteria on the implant surface cannot be eradicated by antibiotics alone, and a procedure without implant exchange may not lead to the desired success (Davies, 2003; Lebeaux et al., 2014). Hence, for chronic infections when infection eradication/cure in a medically fit patient is intended, a revision arthroplasty with exchange of the whole prosthesis is indicated.

In acute PJIs, it is assumed that, although already present, the biofilm is still immature and not fully structured; hence, a DAIR procedure can be effective to eradicate the infection. In general, acute infections are subdivided into early and late acute infections.

#### 3.1.1 Early acute infections

The main cause of an early acute infection is iatrogenic inoculation of microorganisms into the joint or wound at the time of index surgery (Sigmund and McNally, 2019). Occasionally, they can be caused by local or haematogenous (other primary infective foci) spread (Sigmund and McNally, 2019). Patients typically present with an acute onset of symptoms (local: severe pain, oedema, effusion, erythema, increased joint temperature, reduced function ± systemic: pyrexia, tachycardia, sweating, rigours) postoperatively (Sigmund and McNally, 2019). For this group of patients, a low failure rate after a DAIR procedure has been shown in the literature, ranging from 12 % to 35 % (Table 2) (Wouthuyzen-Bakker et al., 2019, 2020b; Shohat et al., 2020; Nurmohamed et al., 2021; Toh et al., 2021; Zhu et al., 2021; Davis et al., 2022; Chang et al., 2022).

**Table 2:** A comparison of the literature between early (EA) and late acute (LA) infections after a DAIR procedure.

Literature	DAIR ( n )	Study design	Early acute (EA)	Late acute (LA)	Failure rate	Failure rate	p value
			definition	definition	EA ( n ; %)	LA ( n ; %)	
Shohat et al. (2020)	1174	Retrospective	Symptoms < 3 weeks	Symptoms < 3 weeks	276/790 (35)	129/384 (34)	0.650^*^
	EA: n=790	2005–2017	<3 months after arthroplasty	>3 months after arthroplasty			
	AH: n=384						
Wouthuyzen-Bakker et	264	Retrospective	Symptoms < 3 weeks	Symptoms < 3 weeks	32/132 (24)	72/132 (54)	<0.001
al. (2020)	EA: n=132	2005–2015	<3 months after arthroplasty	>3 months after arthroplasty			
	AH: n=132						
Nurmohamed et al. (2021)	51	Retrospective	Onset of infection within 3 months	Mean days of symptoms 12 d	11/35 (31)	5/16 (31)	1.000
	EA: n=35	2009–2017	after surgery (mean 22 d)	(0–83 d)			
	AH: n=16						
Toh et al. (2021)	106	Retrospective	Symptoms < 3 weeks	Symptoms < 3 weeks	10/33 (30)	22/73 (30)	0.986
	EA: n=33	2003–2018	<3 months after arthroplasty	>3 months after arthroplasty			
	AH: n=73						
Zhu et al. (2021)	230	Retrospective	<4 weeks after TKA	Infections occurring after the	27/83 (33)	61/102 (60)	$<0.0001^{*}$
	EA: n=83	2001–2015		defined “postoperative” period in a			
	AH: n=102			previous well-functioning implant			
	Chronic:			secondary to infection at a remote			
	n=45			site			
Davis et al. (2022)	427	Prospective	<30 d after original arthroplasty	>30 d from original implant,	41/160 (26)	135/267 (51)	$<0.0001^{*}$
	EA: n=160	AH: 2014–2017		within ≤7 d of symptoms and no			
	n=267			evidence of a sinus communicating			
				with the joint space			
Chang et al. (2022)	101	Retrospective	<3 months following initial knee	>3 months of initial arthroplasty	4/34 (12)	19/67 (28)	0.06
	EA: n=34	2005–2019	arthroplasty surgery (mean duration	surgery (mean duration of			
	AH: n=67		of symptoms 6.8 d)	symptoms 8.3 d)			
Sigmund et al. (2024)	176	Retrospective	<4 weeks since index arthroplasty	<3 weeks of symptoms after an	17/67 (25)	18/46 (39)	0.12
	EA: n=67	2010–2020		uneventful postoperative period			
	AH: n=46						

^*^ Chi-squared test.

**Figure 1:**
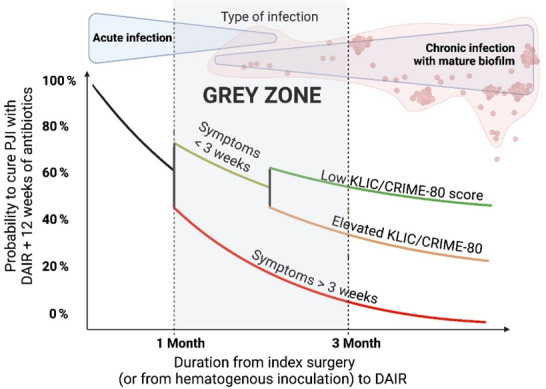
Probability of curing periprosthetic joint infection without consideration of the causing pathogen, its susceptibility pattern, presence of bacteraemia, the soft tissue envelope, the type of surgery, and host and clinical factors.

In the current literature, there are controversies about the definition of an early acute PJI: they are commonly defined as infections occurring within the first 3–4 weeks after the index arthroplasty (primary or revision) (Osmon et al., 2013; Argenson et al., 2019) or as infection within 3 months of the index surgery but less than 3 weeks of symptoms (Argenson et al., 2019; Shohat et al., 2020; Wouthuyzen-Bakker et al., 2020b; Löwik et al., 2020). In the IDSA guidelines (Osmon et al., 2013) and the ICM meeting 2018 (ICM, 2018), early PJI is defined as <30 d after index arthroplasty. When a DAIR procedure was performed in patients with less than 4 weeks following prosthesis implantation, low reinfection rates between 0 %–25 % were reported (Sendi et al., 2017; Clauss et al., 2020; Sigmund et al., 2024). However, Löwik et al. (2020) analysed different time intervals from index arthroplasty to DAIR in their retrospective multicentre study of 769 patients. Although failure rates were higher in comparison to the above-mentioned studies, they demonstrated similar outcomes between 1–2 weeks (failure rate: 42 %, n=95/226 ), 3–4 weeks (38 %, n=143 /378), 5–6 weeks (29 % n=29 /100), and 7–12 weeks (42 %, 27/65) from index arthroplasty. It needs to be highlighted that an exchange of mobile parts was performed in a lower number of patients in the early (1–2 weeks) compared to the late course (7–12 weeks; 41 % vs. 63 %, p<0.001 ), which may have significantly influenced their results.

Due to the current available data, we recommend defining an early infection as <4 weeks following index arthroplasty as well. However, a DAIR procedure can also be done in patients between 4 and 12 weeks after surgery but with symptoms of less than 3 weeks. That said, surgeons need to be aware that the success rate might be reduced in these cases (Fig. 1 – grey zone).

#### 3.1.2 Late acute infections

Late acute PJIs are defined as infections with a sudden onset of symptoms of less than 3 weeks in a prior well-functioning prosthesis and uneventful period more than 3 weeks after implantation (Zimmerli et al., 2004) or as symptoms less than 3 weeks more than 3 months after implantation of the index prosthesis (Wouthuyzen-Bakker et al., 2019). These late acute infections have a different pathogenesis compared to early acute infections. Due to haematogenous spread of bacteria originated from a distant infection source, microorganisms can enter the prosthetic joint, leading to a sudden onset of symptoms (local ± systemic) (Sigmund and McNally, 2019). Rakow et al. (2019) investigated the primary infectious focus in 106 episodes of late acute PJIs. In 72 of the included episodes (68 %), they were able to identify the source, including infected intravascular devices or heart valves (22 episodes), skin and soft tissue infections (16 episodes), oral cavity infections (12 episodes), urogenital infections (12 episodes), gastrointestinal infections (7 episodes), contralateral PJI of the hip (1 episode), pneumonia (1 episode), and epidural abscess and meningitis (1 episode) (Rakow et al., 2019). In a recently published study by Knoll et al. (2023), the most common primary infectious focus was located in the skin and soft tissue ( n=31 /83 episodes), followed by cardiovascular system (28/83), urogenital tract (9/83), gastrointestinal tract (8/83), oral cavity (3/83), respiratory tract (2/83), and bone (2/82). Due to their pathogenesis, late acute infections can occur at any time after index surgery, even decades later. It is crucial to initiate a thorough diagnostic work-up to identify the primary infection source to avoid recurrent infection and seeding to another anatomic site.

Knoll et al. (2023) observed in their univariate analysis that haematogenous origin was associated with a significant higher failure rate compared to non-haematogenous PJIs. Indeed, many authors observed a high failure rate in late acute PJIs after a DAIR procedure, ranging from 28 % to 60 % (Wouthuyzen-Bakker et al., 2019, 2020b; Shohat et al., 2020; Nurmohamed et al., 2021; Toh et al., 2021; Zhu et al., 2021; Davis et al., 2022; Chang et al., 2022). In some studies, a statistically significant lower success rate was demonstrated in late acute PJIs compared to early acute PJIs (Table 2) (Wouthuyzen-Bakker et al., 2020b; Zhu et al., 2021; Davis et al., 2022). The high failure rate may be explained by the difficulty to determine the accurate starting point of symptoms potentially misdiagnosing a chronic infection as acute. Some PJIs (especially caused by low-virulence organisms such as CoNS or *Cutibacterium* spp.) classified as “late acute” may represent an acute flare up or exacerbation of a chronic infection, making an accurate diagnosing challenging. Another explanation may be continuous seeding of microorganisms to the prosthetic joint from the still existing infection source (e.g. endocarditis or another intravascular infection), leading to a reinfection of the periprosthetic joint (although the prior DAIR procedure would have been successful). Additionally, a higher bacterial inoculum in late acute infections in comparison to early acute PJIs may be possible. Finally, it may be a late acute superinfection of a previously undetected low-grade infection. However, the exact reason for the high failure rate is still unknown.

Nevertheless, Shohat et al. (2020) and Toh et al. (2021) were not able to calculate a different failure rate between early and late acute infections in their studies, including a reasonable number of cases (Table 2). The lack of a uniform haematogenous PJI definition prevents accurate comparison between studies and institutions. The optimal cut-off time of symptom duration to distinguish between acute and chronic has not been determined in comparative studies yet and is still a matter of debate. Furthermore, the cut-off time until when a DAIR can be successfully performed is still controversial.

However, the time interval between symptom onset and DAIR procedure is strongly associated with DAIR success (Karczewski et al., 2019; Kuiper et al., 2013; Kunutsor et al., 2018; Sendi et al., 2017). In the ICM guideline, a symptom duration of <4 weeks (favourable 7 d) was recommended (Argenson et al., 2019). The IDSA advocated <3 weeks from symptom onset to surgical treatment (Osmon et al., 2013), which is the same cut-off as recommended by Zimmerli et al. (2004). Many authors tried to find the optimal cut-off until when a DAIR procedure can be done successfully. Qu et al. (2019) demonstrated in their review and pooling analysis of 33 studies including 1266 periprosthetic knee infections a significantly improved success rate of DAIR when the time from symptoms to debridement was less than 3 weeks. The overall pooled mean success rate was 57 % in their analysis. When only studies with a mean symptom duration of <3 weeks were considered, a pooled success rate of 71 % was calculated, while it was only 35 % in studies with a mean symptom duration of >3 weeks ( p<0.05 ) (Qu et al., 2019). Kunutsor et al. (2018) showed similar results in their meta-analysis of 93 articles including 4897 PJIs treated with DAIR. Although not significant, a shorter duration between onset of symptoms to DAIR of less than 21 d had a higher success rate compared to a longer duration ( ≥21 d) in their study ( p=0.071 ). Tsang et al. (2017) were also able to demonstrate an association between onset of symptoms to DAIR and success in their review of 39 studies involving 1296 patients with periprosthetic hip infections. Although no difference ( p=0.215 ) was observed between studies with a median symptom duration of <4 weeks (73 %, 354/485 patients) and >4 weeks (69 %, 375/540 patients), an improved survival was demonstrated when the cut-off time from symptom onset to debridement was reduced to 7 d. In studies with a median symptom duration of <7 d (198/275 patients), a pooled success rate of 72 % was calculated, while it was only 52 % in studies with a median symptom duration of >7 d (170/329 patients) ( p<0.0001 ) (Tsang et al., 2017). Many other authors observed similar results with a better outcome using a symptom duration of <7 d (Urish et al., 2018; Grammatopoulos et al., 2017a; Koh et al., 2015; Kuiper et al., 2013; Marculescu et al., 2006).

However, in some studies (including a smaller sample size), no difference was seen between a symptom duration of <7 and >7 d (Swenson et al., 2018; Grammatopoulos et al., 2017b). In a retrospective study of 78 periprosthetic knee infections by Triantafyllopoulos et al. (2015), symptom duration of >5 d ( n=33 /78) was found to be an independent predictor of I&D failure with a 95 % lower odds of success compared with patients with ≤5 d of symptoms ( n=45 /78) ( p<0.0001 ). Furthermore, the odds of success decreased by 7.5 % for each additional day of symptom duration in their logistic regression analysis (OR = 0.925, 95 % CI: 0.862–0.993; p=0.0312 ) (Triantafyllopoulos et al., 2015). Even a cut-off time of ≤2 d for a successful DAIR was reported in the literature (Brandt et al., 1997; Fink et al., 2017). Bedair et al. (2020) recommended in their more recently published multicentre retrospective study of 316 patients undergoing DAIR for PJI to perform a DAIR immediately to achieve the best outcomes. However, they were not able to calculate a statistically significant difference between patients treated within 2 d of symptom onset (infection control rate: 60 %) and patients treated between 3 and 7 d (53 %) ( p=0.23 ).

#### Recommendation

Early acute PJI is defined as infection occurring within the first 4 weeks after index arthroplasty and late acute PJI as patients with <3 weeks of symptoms after an uneventful postoperative period and >4 weeks after index arthroplasty. In general, it is recommended that a DAIR procedure should be done timely in case of an acute PJI. We recommend to perform a DAIR procedure urgently within 3 weeks of symptoms, preferably within the first 7 d. However, the procedure should not be seen as an emergency (done at night by an unexperienced non-infection surgeon, mobile parts for a specific prosthesis may not be available on admission day).

In Fig. 1, the probability of curing PJI is illustrated without consideration of the causing pathogen, its susceptibility pattern, presence of bacteraemia, the soft tissue envelope, the type of surgery, and host and clinical factors.

### 3.2 Type of surgery

#### 3.2.1 Total hip vs. total knee arthroplasty

The impact of the arthroplasty site (hip vs. knee) on DAIR outcome is still a matter of debate (Table 3). In a review and meta-regression analysis of 65 studies comprising 6630 patients, higher success rates were reported in hips compared to knees (70 %, 95 % CI: 65 %–75 %; vs. 63 %, 95 % CI: 58 %–69 %) (Gerritsen et al., 2021). Similar results were described in another meta-analysis including 93 articles (4897 PJIs) by Kunutsor et al. (2018). In this study, the success rate following DAIR in THA was 75 % (95 % CI: 69 %–82 %) and in TKA 53 % (95 % CI: 45 %–60 %, p<0.001 ) (Kunutsor et al., 2018). The worse outcome after a DAIR in knees may be explained by the fact that in some of the mentioned studies arthroscopic knee washouts were included. However, Bernaus et al. (2022) observed a higher failure rate in knee patients with an early acute PJI treated with an open debridement (15 % vs. 9 %, p=0.049 ) as well. Explanations for the higher failure rate in prosthetic knee joints may be the inferior soft tissue coverage, different blood supply, and/or a larger implant surface susceptible to bacterial attachment compared to the hip. However, other more recently published studies showed no difference between hip and knee DAIRs (Table 3) (Zmistowski et al., 2016; Löwik et al., 2018; Wouthuyzen-Bakker et al., 2019; Shohat et al., 2020; Nurmohamed et al., 2021). Hence, further studies with modern DAIR treatment strategies are needed to analyse this topic.

**Table 3:** A comparison of the literature of DAIR failure rates based on the affected joint.

Literature	Study design	DAIR ( n )	Type of	Failure rate	Failure rate	p value
			infection	hip	knee	
Byren et al. (2009)	Retrospective	112	All PJIs	7/52 (13)	13/51 (25)	0.123^*^
Zmistowski et al. (2016)	Retrospective	153	NA	26/60 (43)	47/93 (51)	0.41
Löwik et al. (2018)	Retrospective	386	EA	115/296 (39)	32/86 (37)	0.697
Wouthuyzen-Bakker et al. (2019)	Retrospective	340	LA	46/93 (49)	107/247 (43)	0.31
Shohat et al. (2020)	Retrospective	1174	EA, LA	208/565 (37)	197/609 (32)	0.111
Nurmohamed et al. (2021)	Retrospective	67	EA, LA	12/41 (29)	11/26 (42)	0.273^*^
Bernaus et al. (2022)	Retrospective	376	EA	18/206 (9)	26/170 (15)	0.049

EA – early acute infection, LA – late acute infection, NA – not available. ^*^ Chi-squared test.

#### Recommendation

Based on the current literature, a DAIR can be done in THA as well as TKA.

#### 3.2.2 Primary vs. revision arthroplasty

DAIR failure rates following primary total joint replacement ranged between 13 % and 50 % and after revision arthroplasty between 11 % and 54 % in the literature (Table 4) (Byren et al., 2009; Lora-Tamayo et al., 2013, 2017; Triantafyllopoulos et al., 2015; Zmistowski et al., 2016; Grammatopoulos et al., 2017b; Löwik et al., 2018; Wouthuyzen-Bakker et al., 2019; Shohat et al., 2020; Nurmohamed et al., 2021; Chang et al., 2022; Bernaus et al., 2022). Some authors showed a lower DAIR success rate in patients with previously revised joints ( p<0.05 ) (Byren et al., 2009; Wouthuyzen-Bakker et al., 2019; Lora-Tamayo et al., 2017; Tornero et al., 2015). Indeed, Byren et al. (2009) found a significant increased risk of DAIR failure with a HR of 3.1 (95 % CI: 1.2–8.3, p=0.008 ) when patients had previous revision surgeries. Due to an OR of 4.3 (95 % CI: 1.3–14.0), Tornero et al. (2015) included revision surgery as an independent predictor of DAIR failure in their KLIC score for early acute infections. Nurmohamed et al. (2021) reported a higher infection control rate in patients undergoing a DAIR procedure after primary arthroplasty (69 %, n=35 /51) compared to patients treated with DAIR after PJI-related revision arthroplasty (56 %, n=9 /16), but no statistically significant difference was observed ( p=0.363 ) and the sample size was small. In addition, other authors were also not able to find an association between the arthroplasty type (primary vs. revision) and DAIR outcome (Lora-Tamayo et al., 2013; Triantafyllopoulos et al., 2015; Zmistowski et al., 2016; Grammatopoulos et al., 2017b; Löwik et al., 2018; Shohat et al., 2020; Chang et al., 2022; Bernaus et al., 2022). Bernaus et al. (2022) observed a higher failure rate in the primary arthroplasty group (14 %) compared to the revision arthroplasty group (11 %) in their large multicentre study of 455 early acute PJIs ( p=0.402 ). They explained their controversial results and better outcome by the fact that DAIR in the revision group was more frequently performed at specialized infection units and specialized revision arthroplasty surgeons (Bernaus et al., 2022).

**Table 4:** A comparison of the literature of DAIR failure rates following primary or revision arthroplasty.

Literature	DAIR ( n )	Study design	Type of	Failure rate	Failure rate	p value
			infection	primary	revision	
Byren et al. (2009)	112	Retrospective	All PJIs	11/86 (13)	9/26 (35)	0.011^*^
Lora-Tamayo et al. (2013)	345	Retrospective	EA, LA	111/264 (42)	34/64 (53)	0.109^*^
Zmistowski et al. (2016)	131	Retrospective	NA	55/111 (50)	13/20 (65)	0.203^*^
Grammatopoulos et al. (2017b)	122	Retrospective	All PJIs	26/82 (32)	14/40 (35)	0.716
Lora-Tamayo et al. (2017)	444	Retrospective	EA, LA	127/332 (38)	60/112 (54)	0.005^*^
Löwik et al. (2018)	386	Retrospective	EA	123/330 (37)	25/56 (45)	0.294
Wouthuyzen-Bakker et al. (2019)	340	Retrospective	LA	101/242 (42)	52/96 (54)	0.04
Shohat et al. (2020)	1174	Retrospective	EA, LA	309/923 (33)	96/251 (38)	0.182
Nurmohamed et al. (2021)	67	Retrospective	EA, LA	16/51 (31)	7/16 (44)	0.363^*^
Chang et al. (2022)	101	Retrospective	EA, LA	19/90 (21)	4/12 (33)	0.462
Bernaus et al. (2022)	455	Retrospective	EA	15/108 (14)	29/268 (11)	0.402

EA – early acute infection, LA – late acute infection. ^*^ Chi-squared test. NA – not available.

#### Recommendation

Patients with previous revision(s) were associated with worse outcomes following DAIR in some studies. This may be due to soft tissue disruption, scar formation, and reduced blood supply after additional surgeries but also due to a missed chronic PJI during the previous so-called aseptic revision. Hence, this factor should be considered in the decision-making process prior to surgical treatment. A multidisciplinary team approach in a specialized infection centre may be needed to achieve the best outcome in these cases.

#### 3.2.3 Megaprostheses for non-oncological conditions

A particular challenge for arthroplasty surgeons is an infection after megaprosthesis reconstruction in patients with major bone loss (septic or aseptic) or periprosthetic fracture. Due to prolonged operation times, larger implant surfaces (susceptible to bacterial attachment), and a frequently poor soft tissue envelope, an increased risk of periprosthetic infection is present (Sukhonthamarn et al., 2021; Argenson et al., 2019; Alvand et al., 2018). Alvand et al. (2018) analysed the outcomes of 69 megaprostheses performed for PJI treatment following complex arthroplasty (n = 45), failed osteosynthesis ( n=12 ), failed multiple DAIRs ( n=7 ), and periprosthetic fracture ( n=5 ). An overall 48 % complication rate was observed. PJI recurrence was reported in 19 patients (28 %) who all needed further surgery (DAIR n=5 , revision of megaprosthesis n=5 , above knee amputation n=1 ) or life-long suppressive antibiotics (Alvand et al., 2018). However, little is known about the effectiveness of a DAIR procedure in infected megaprosthesis for non-oncological patients. In a cohort of 14 patients undergoing DAIR for infection of a megaprosthesis (6 proximal femoral replacements (PFRs), 5 distal femoral replacements (DFRs), 3 total femoral replacements), 7 patients (50 %) were treated successfully with a single DAIR and 2 needed a second DAIR procedure (14 %) (Asokan et al., 2022). In the remaining five patients (36 %), an exchange of the prosthesis (two-stage: n=4 , one-stage: n=1 ) was required (Asokan et al., 2022). In another cohort of 33 infected megaprostheses, including 15 PFRs (45 %) and 18 DFRs (55 %), DAIR was performed in 27 patients (82 %) consisting of 11 early acute, 9 late acute, and 7 chronic PJIs (Sukhonthamarn et al., 2021). The overall success rate of DAIR was 63 %. When a modular component exchange was performed, a higher treatment success was seen (68 % vs. 50 %) but not at a significant level ( p>0.05 ). Treatment success in early acute PJIs was 82 % ( n=9 /11), in late acute PJIs 44 % ( n=4 /9), and in chronic PJIs 57 % ( n=4 /7) (Sukhonthamarn et al., 2021). A single DAIR was only successful in 30 % of patients. Two DAIR procedures which were done in 53 % of their patients showed a success rate of 44 %, and ≥3 DAIRs had a success rate of only 25 % in their study (Sukhonthamarn et al., 2021). However, the number of included cases in both mentioned studies is low.

#### Recommendation

Due to the available data and often limited treatment options, a DAIR procedure with exchange of the modular components and thorough debridement may be considered a treatment strategy in some of the infected megaprostheses, especially in early acute PJIs. However, there is still a lack of studies comparing various treatment strategies (DAIR, one-stage, two-stage, suppressive therapy). Thus, an individual multidisciplinary team discussion considering all host and clinical factors is of utmost importance in these challenging cases.

**Table 5:** A comparison of the literature of the overall success rates after two DAIR procedures.

Literature	DAIR ( n )	Study design	Type of	Failure rate	Failure rate	Overall success
			infection	first DAIR	second DAIR	rate 2 DAIRs
Triantafyllopoulos et al. (2016)	141	Retrospective	EA, LA	44/122 (36)	9/19 (47)	88/141 (62)
Grammatopoulos et al. (2017a)	82	Retrospective	All PJIs	26/82 (32)	6/20 (30)	70/82 (85)
Grammatopoulos et al. (2017b)	122	Retrospective	All PJIs	39/122 (32)	11/32 (34)	104/122 (85)
Urish et al. (2018)	216	Retrospective	All PJIs	109/216 (51)	24/59 (41)	142/216 (66)
Wouthuyzen-Bakker et al. (2020a)	455	Retrospective	EA, LA	144/455 (32)	37/144 (26)	418/455 (92)

EA – early acute infection, LA – late acute infection.

#### 3.2.4 Additional DAIR after failed initial debridement

Performance of a second or multiple DAIRs in the event of a failed initial DAIR is still a matter of debate in the literature (Table 5) (Wouthuyzen-Bakker et al., 2020a; Triantafyllopoulos et al., 2016; Urish et al., 2018; Grammatopoulos et al., 2017a, b). In the last ICM guideline a recommendation against a second or multiple DAIRs was given based on the available studies in 2018 (Argenson et al., 2019). Indeed, Triantafyllopoulos et al. (2016) reported a 47 % failure rate ( n=9 /19) when DAIR was repeated in their retrospective cohort (Table 5). Additionally, an interval of >20 d between DAIRs had 97 % lower odds of success (implant retention) than <20 d (OR: 0.026, 95 % CI: 0.001–0.934, p=0.046 ) (Triantafyllopoulos et al., 2016). Hence, the authors concluded that a second or multiple DAIRs should be avoided. In a multicentre cohort study of 216 PJIs of the knee, 109 cases (51 %) failed after the initial DAIR procedure (Urish et al., 2018). Of these, 59 underwent a second DAIR and 24 ( n=24 /59, 41 %) failed again. In the end, only 28 % of the initially failed TKA cases had a DAIR as final procedure (Urish et al., 2018). The study group concluded that if the initial operation fails, an additional DAIR has a low probability of success. Other authors also reported a higher rate of failure when ≥2 debridements were necessary ( p<0.05 ) (Lora-Tamayo et al., 2013, 2017). In addition, Toh et al. (2021) showed a strong correlation between repeat DAIR and failure (OR 5.27, 95 % CI: 1.99–13.9, p<0.01 ) in their more recently published retrospective study of 106 acute PJIs. Of the 24 cases requiring a second debridement, only 9 cases (38 %) were successfully treated, and no further intervention was required. Based on these results, they recommended a two-stage revision in case of a failed DAIR procedure (Toh et al., 2021).

However, Wouthuyzen-Bakker et al. (2020a) showed in a large series of 455 acute PJI cases managed by DAIR a lower failure rate of only 26 % after a second DAIR. Of the 455 initially treated DAIRs, 144 (32 %) failed and underwent a second procedure. Of these, only 37 cases ( n=37 /144, 26 %) failed again. The lower failure rate may be explained by the fact that 62 % of the second DAIRs had negative cultures. In their multivariant analysis, only chronic renal failure (OR 13.6, 95 % CI: 2.03–91.33, p=0.007 ) and positive cultures collected intraoperatively during the second DAIR (OR 3.16, 95 % CI: 1.29–7.74, p=0.01 ) were independent predictors of failure. Due to their good results, the authors stated “a second DAIR should not be discarded in acute PJIs” (Wouthuyzen-Bakker et al., 2020a). However, it should be noted that the clinical threshold of performing a second DAIR in this cohort ( ±30 % after the initial DAIR) was lower compared to other studies, which is reflected in the high percentage of negative cultures during the second DAIR procedure.

Overall, success rates after a second DAIR were moderate to good and ranged between 53 % and 74 % in the literature (Table 5) (Triantafyllopoulos et al., 2016; Wouthuyzen-Bakker et al., 2020a). However, these results need to be viewed with caution. Most studies included a low number of cases, were heterogenous (e.g. type of infection, indication), and provided limited information about their indication for a second DAIR. In addition, there is still a lack of studies comparing various treatment strategies after a failed first DAIR.

#### Recommendation

A second DAIR procedure may be considered for certain patients where the implant is well fixed, and the soft tissue envelope is intact, especially if the first debridement was unsatisfactory and/or no mobile parts were exchanged. When the second DAIR is performed based on signs and symptoms suggesting microbiological failure, removal of the implant should be strongly considered when infection cure is still intended.

### 3.3 Soft tissue envelope

In general, a DAIR procedure is recommended in patients with a good soft tissue envelope and adequate bone stock without the presence of a sinus tract (Osmon et al., 2013; Zimmerli et al., 2004; Argenson et al., 2019; Byren et al., 2009). Hence, many authors already exclude patients with a sinus tract in their studies; however, others do not consider a sinus tract a contraindication as long as the implant is stable.

**Table 6:** A comparison of the failure rates in the literature between patients with and without a sinus tract in PJI cases after a DAIR procedure.

Literature	DAIR ( n )	Study design	Type of	Failure	Failure	p value
			infection	no sinus	sinus tract	
Tornero et al. (2015)	222	Retrospective	EA	47/205 (23)	5/17 (29)	0.761
Lora-Tamayo et al. (2017)	444	Retrospective	EA, LA	155/378 (41)	27/61 (44)	0.58
Löwik et al. (2018)	386	Retrospective	EA	139/345 (40)	9/41 (22)	0.022
Shohat et al. (2020)	1174	Retrospective	EA, LA	315/872 (36)	90/302 (30)	0.049
Deng et al. (2021)	107	Retrospective	EA, LA, C	15/55 (27)	19/52 (37)	0.304^*^

EA – early acute infection, LA – late acute infection, C – chronic. ^*^ Chi-squared test.

#### 3.3.1 Sinus tract

The impact of a sinus tract on DAIR outcome is still unclear (Table 6). Some studies reported a higher failure rate in patients with a sinus tract (Marculescu et al., 2006; Lora-Tamayo et al., 2013). Lora-Tamayo et al. (2013), for example, observed a higher risk of late failure in cases with a sinus tract in their multivariate analysis of 345 cases with *S. aureus* PJIs (OR 2.28, 95 % CI: 1.13–4.21, p=0.029 ). However, multiple other studies showed no correlation between the presence of a sinus tract and DAIR failure (Table 6) (Lora-Tamayo et al., 2017; Tornero et al., 2015; Deng et al., 2021). For instance, Deng et al. (2021) concluded that a sinus tract does not influence DAIR success. Although a higher failure rate was observed in their study including 107 PJI cases (sinus tract ( n=52 ): 37 % vs. no sinus tract ( n=55 ): 27 %), the difference was not statistically significant ( p=0.304 ). They additionally observed a better outcome in these patients when a modular component exchange was done (76 % vs. 47 %, p=0.038 ). Interestingly, two studies found a lower failure rate in patients with a sinus tract (Table 6) (Shohat et al., 2020; Löwik et al., 2018).

Hence, there are fundamental inconsistencies in the literature. These discrepancies may be explained by the unclear definition of a sinus tract.

The clinical definition of a sinus tract is a communication from the joint cavity, prosthesis, or periprosthetic tissue to the skin surface caused by the increasing pressure originated from the infectious exudate. A sinus tract is defined as having an epithelium and an established tract/canal to the prosthesis. This is typical for chronic infections. However, in early acute infections, the wound is generally not completely closed, and even low pressure from the infectious exudate can re-open the wound (wound leakage, dehiscence, or breakdown, which is not the same as a chronic sinus tract). A potential explanation for the literature discrepancy is that the presence of a sinus tract in the articles with good outcomes was indeed a wound dehiscence/breakdown, and in those with worse outcomes, it was a real sinus tract in a chronic infection.

#### 3.3.2 Persistent wound drainage

In the early acute period, patients can develop postoperative drainage. Due to the operation and more permeable soft tissue, less joint fluid pressure is needed to form a channel to the outside environment. In a study of 10 325 patients undergoing total hip and knee arthroplasty (11 785 cases), 300 patients (3 %) suffered from persistent wound drainage ( >48 h) (Jaberi et al., 2008). In 217 patients (72 %), it stopped between day 2 and 4. None of these patients needed further intervention, and all these patients were infection-free at the 1-year follow-up. In the remaining 83 patients (28 %), further surgery was necessary, but only 34 % ( n=28 /83) showed positive cultures intraoperatively. However, all patients with persistent wound drainage received prophylactic oral antibiotics (cephalexin or clindamycin) while drainage was present. Hence, the results need to be interpreted with caution. A multicentre prospective observational study evaluated the amount and duration of postoperative wound drainage in the first 4 weeks following total joint arthroplasty in 1019 patients (46 % THA, 54 % TKA) (Scheper et al., 2023). Any wound drainage in week 2 (OR 50.83, 95 % CI: 95 % CI: 11.41–226.51), moderate to severe wound drainage in the third week (OR 103.23, 95 % CI: 26.08–408.57), newly developed wound drainage in week 2 (after a week without drainage, OR 80.71, 95 % CI: 9.12–714.52), and more than 5 cumulative days of wound drainage during the first 3 weeks (OR 9.20, 95 % CI: 3.37–25.14) showed a strong association with PJI. Sensitivity and specificity of drainage > 5 d during the first 3 weeks were 63 % and 87 %, respectively. In another study of 1181 TKAs and 1124 THAs, >5 d of postoperative wound drainage significantly increased the risk of infection (Saleh et al., 2002). Another study found that each day of persistent drainage is associated with an increased risk of infection (THA: 42 %, TKA: 29 %) (Patel et al., 2007). A prospective randomized controlled trial is currently being conducted to find the optimal way to manage wound drainage following THA and TKA (Löwik et al., 2017).

#### 3.3.3 Other soft tissue complications

In late acute infections, when the wound initially healed uneventfully and the patient was symptom-free, a sudden onset of symptoms can be followed by a draining sinus. However, sinus tract formation can take some time when the soft tissues are intact, and, hence, it can be assumed that the microbial biofilm maturation is already completed, and a chronic infection is most likely present. Surgery with exchange of implants is recommended in these cases. In some rare late acute infections, the soft tissues are compromised, and a sinus tract can occur very quickly. In these cases, an exchange of the prosthesis can be recommended as well due to the poor soft tissue envelope. A staged revision should also be considered in early acute infections with major soft tissue defects requiring muscle flap reconstructions. Additionally, chronic PJI patients can have a flare-up of their infection, and an immature draining sinus tract can occur. In these cases, an exchange of the prosthesis is recommended due to the chronic course of the disease.

#### Recommendation

The presence of a sinus tract and/or compromised soft tissues can be seen as contraindications for a DAIR procedure. Due to the results in the literature, it seems that a prolonged wound drainage of >5 d is associated with infection, and, hence, a DAIR procedure can be recommended in these cases.

### 3.4 Host-related and clinical factors

Various host-related and clinical factors were described as independent predictors of DAIR failure in the literature (Table 7) (Löwik et al., 2018; Davis et al., 2022; Triantafyllopoulos et al., 2015; Zmistowski et al., 2016; Swenson et al., 2018; Shohat et al., 2020; Zhu et al., 2021; Chang et al., 2022; Veerman et al., 2022b; Bernaus et al., 2022; Lora-Tamayo et al., 2017; Wouthuyzen-Bakker et al., 2019; Nurmohamed et al., 2021; Toh et al., 2021; Grammatopoulos et al., 2017a, b). In the past, such factors were not fully considered in the decision-making process for a DAIR procedure (Osmon et al., 2013), but existing comorbidities may increase the risk of complications and failure (Wouthuyzen-Bakker et al., 2021). Indeed, Davis et al. (2022) showed an association between the presence of at least one comorbidity and treatment failure in their prospective multicentre cohort study of 653 PJI patients (OR 0.43, 95 % CI: 0.27–0.67; p<0.001 ). Therefore, the most analysed and important host-related and clinical factors are discussed based on the available literature.

**Table 7:** A comparison of the literature of important host factors (age, sex, rheumatoid arthritis (RA), immunosuppressive therapy (IT), body mass index (BMI), diabetes, chronic kidney failure, liver disorder, chronic obstructive pulmonary disease (COPD), nicotine use (smoking), serum C-reactive protein (CRP), American Society of Anesthesiologists (ASA)).

Age	DAIR ( n )	Study design	Type of	Success	Failure	p value
			infection	mean	mean	
				age (SD)	age (SD)	
Löwik et al. (2018)	386	Retrospective	EA	72 (11)	75 (11)	0.009
Triantafyllopoulos et al. (2015)	78	Retrospective	EA, LA	65 (10)	64 (11)	0.845
Zmistowski et al. (2016)	153	Retrospective	NA	64 (–)	64 (–)	0.85
Swenson et al. (2018)	72	Retrospective	EA, LA	67 (–)	61 (–)	0.066
Shohat et al. (2020)	1174	Retrospective	EA, LA	70 (11)	71 (13)	0.432
Zhu et al. (2021)	230	Retrospective	EA, LA, C	68 (10)	68 (11)	0.833
Chang et al. (2022)	101	Retrospective	EA, LA	74 (9)	73 (8)	0.679
Veerman et al. (2022b)	88	Retrospective	EA	66 (11)	66 (11)	0.304
Bernaus et al. (2022)	376	Retrospective	EA	71 (12)	72 (9)	0.802
Sex	DAIR	Study design	Type	Success	Failure	p value
	(male)		infection	male ( n , %)	male ( n , %)	
Shohat et al. (2020)	1174 (538)	Retrospective	EA, LA	334/769 (43)	204/405 (50)	0.027
Triantafyllopoulos et al. (2015)	78 (37)	Retrospective	EA, LA	21/43 (49)	16/35 (46)	0.784
Zmistowski et al. (2016)	153 (61)	Retrospective	NA	35/80 (44)	26/73 (36)	0.33
Lora-Tamayo et al. (2017)	444 (219)	Retrospective	EA, LA	122/257 (47)	97/187 (52)	0.30
Löwik et al. (2018)	386 (148)	Retrospective	EA	79/238 (33)	69/148 (47)	0.08
Swenson et al. (2018)	72 (35)	Retrospective	EA, LA	26/53 (49)	9/19 (47)	1.000
Wouthuyzen-Bakker et al. (2019)	340 (175)	Retrospective	LA	89/187 (48)	86/153 (56)	0.11
Nurmohamed et al. (2021)	67 (29)	Retrospective	EA, LA	16/44 (36)	13/23 (57)	0.114^*^
Toh et al. (2021)	106 (41)	Retrospective	EP, AH	26/74 (35)	15/32 (47)	0.255
Zhu et al. (2021)	230 (142)	Retrospective	EP, AH, C	75/124 (60)	67/106 (63)	0.672
Veerman et al. (2022b)	88 (48)	Retrospective	EA	36/60 (60)	12/28 (43)	0.133
Bernaus et al. (2022)	376 (169)	Retrospective	EA	150/332 (45)	19/44 (43)	0.802
Chang et al. (2022)	101 (20)	Retrospective	EA, LA	15/78 (19)	5/23 (22)	0.772
Rheumatoid arthritis	DAIR ( n )	Study design	Type of	Success –	Failure –	p value
			infection	RA ( n , %)	RA ( n , %)	
Lora-Tamayo et al. (2017)	444	Retrospective	EA, LA	13/257 (5)	24/187 (13)	<0.01
Wouthuyzen-Bakker et al. (2019)	340	Retrospective	LA	7/187 (4)	20/153 (13)	0.001
Shohat et al. (2020)	1174	Retrospective	EA, LA	46/769 (6)	39/405 (10)	0.025
Zmistowski et al. (2016)	153	Retrospective	NA	2/80 (3)	5/73 (7)	0.26
Löwik et al. (2018)	386	Retrospective	EA	17/238 (7)	11/148 (7)	0.915
Swenson et al. (2018)	72	Retrospective	EA, LA	4/53 (8)	0/19 (0)	0.517
Zhu et al. (2021)	230	Retrospective	EA, LA, C	9/124 (7)	9/106 (9)	0.729
Veerman et al. (2022b)	88	Retrospective	EA	6/60 (10)	6/28 (21)	0.146
Immunosuppressive therapy	DAIR ( n )	Study design	Type of	Success	Failure	p value
(IT)			infection	IT ( n , %)	IT ( n , %)	
Lora-Tamayo et al. (2017)	444	Retrospective	EA, LA	19/257 (7)	29/187 (16)	<0.01
Wouthuyzen-Bakker et al. (2019)	340	Retrospective	LA	15/187 (8)	24/153 (16)	0.03
Shohat et al. (2020)	1174	Retrospective	EA, LA	75/769 (10)	63/405 (16)	0.004
Veerman et al. (2022b)	88	Retrospective	EA	2/60 (3)	6/28 (21)	0.012
Swenson et al. (2018)	72	Retrospective	EA, LA	4/53 (8)	0/19 (0)	0.517
Zhu et al. (2021)	230	Retrospective	EA, LA, C	7/124 (6)	7/106 (7)	0.762
BMI	DAIR ( n )	Study design	Type of	Success	Failure	p value
			infection	mean BMI	mean BMI	
				(SD)	(SD)	
Triantafyllopoulos et al. (2015)	78	Retrospective	EA, LA	32 (8)	34 (8)	0.369
Zmistowski et al. (2016)	153	Retrospective	NA	33 (–)	34 (–)	0.59
Swenson et al. (2018)	72	Retrospective	EA, LA	36 (–)	35 (–)	0.898

**Table 7:** Continued.

BMI	DAIR ( n )	Study design	Type of	Success	Failure	p value			
			infection	mean BMI	mean BMI				
				(SD)	(SD)				
Löwik et al. (2018)	386	Retrospective	EA	30 (7)	29 (6)	0.117			
Shohat et al. (2020)	1174	Retrospective	EA, LA	31 (7)	31 (6)	0.267			
Zhu et al. (2021)	230	Retrospective	EA, LA, C	34 (16)	33 (16)	0.734			
Veerman et al. (2022b)	88	Retrospective	EA	30 (5)	31 (7)	0.548			
Chang et al. (2022)	101	Retrospective	EA, LA	27 (5)	26 (4)	0.381			
Diabetes	DAIR ( n )	Study design	Type of	Success	Failure	p value			
			infection	diabetes	diabetes				
				( n , %)	( n , %)				
Triantafyllopoulos et al. (2015)	78	Retrospective	EA, LA	12/43 (28)	4/35 (11)	0.073			
Zmistowski et al. (2016)	153	Retrospective	NA	13/80 (16)	13/73 (18)	0.83			
Lora-Tamayo et al. (2017)	444	Retrospective	EA, LA	58/257 (23)	50/187 (27)	0.38			
Löwik et al. (2018)	386	Retrospective	EA	46/238 (19)	36/148 (24)	0.243			
Swenson et al. (2018)	72	Retrospective	EA, LA	15/53 (28)	7/19 (37)	0.687			
Wouthuyzen-Bakker et al. (2019)	340	Retrospective	LA	43/187 (23)	42/153 (28)	0.49			
Shohat et al. (2020)	1174	Retrospective	EA, LA	146/769 (19)	95/405 (24)	0.080			
Toh et al. (2021)	106	Retrospective	EA, LA	14/74 (19)	5/32 (16)	0.684			
Zhu et al. (2021)	230	Retrospective	EA, LA, C	27/124 (22)	22/106 (21)	0.851			
Veerman et al. (2022b)	88	Retrospective	EA	9/60 (15)	2/28 (7)	0.491			
Chang et al. (2022)	101	Retrospective	EA, LA	37/78 (47)	11/23 (48)	0.974			
Chronic kidney failure	DAIR ( n )	Study design	Type of	Success	Failure	p value			
			infection	– renal	– renal				
				failure	failure				
				( n , %)	( n , %)				
Tornero et al. (2015)	222	Retrospective	EA	8/170 (5)	12/52 (23)	<0.001			
Bernaus et al. (2022)	376	Retrospective	EA	34/332 (10)	9/44 (21)	0.045			
Triantafyllopoulos et al. (2015)	78	Retrospective	EA, LA	2/43 (5)	4/35 (11)	0.399			
Lora-Tamayo et al. (2017)	444	Retrospective	EA, LA	20/257 (8)	24/187 (13)	0.079			
Löwik et al. (2018)	386	Retrospective	EA	15/238 (6)	11/148 (7)	0.667			
Wouthuyzen-Bakker et al. (2019)	340	Retrospective	LA	16/187 (9)	10/153 (7)	0.49			
Shohat et al. (2020)	1174	Retrospective	EA, LA	53/769 (7)	36/405 (88)	0.246			
Zhu et al. (2021)	230	Retrospective	EA, LA, C	12/124 (10)	10/106 (9)	0.950			
Liver cirrhosis	DAIR ( n )	Study design	Type of	Success	Failure	p value			
			infection	– liver	– liver				
				disease	disease				
				( n , %)	( n , %)				
Tornero et al. (2015)	222	Retrospective	EA	12/170 (7)	11/52 (21)	0.004			
Triantafyllopoulos et al. (2015)	78	Retrospective	EA, LA	3/43 (7)	1/35 (3)	0.623			
Löwik et al. (2018)	386	Retrospective	EA	1/238 (0)	3/148 (2)	0.130			
Wouthuyzen-Bakker et al. (2019)	340	Retrospective	LA	5/187 (3)	6/153 (4)	0.52			
Shohat et al. (2020)	1174	Retrospective	EA, LA	21/769 (3)	20/405 (5)	0.065			
Bernaus et al. (2022)	376	Retrospective	EA	17/332 (5)	4/44 (9)	0.289			
COPD	DAIR ( n )	Study design	Type of	Success	Failure	p value			
			infection	COPD	COPD				
				( n , %)	( n , %)				
Shohat et al. (2020)	1174	Retrospective	EA, LA	104/769 (14)	72/405 (18)	0.048			
Tornero et al. (2015)	222	Retrospective	EA	27/170 (16)	7/52 (14)	0.671			
Löwik et al. (2018)	386	Retrospective	EA	43/238 (18)	38/148 (26)	0.074			
Wouthuyzen-Bakker et al. (2019)	340	Retrospective	LA	15/187 (8)	19/153 (12)	0.18			
Zhu et al. (2021)	230	Retrospective	EA, LA, C	5/124 (4)	4/106 (4)	1.000			

**Table 7:** Continued.

Nicotine use (smoking)	DAIR ( n )	Study design	Type of	Success	Failure	p value			
			infection	smoking	smoking				
				( n , %)	( n , %)				
Triantafyllopoulos et al. (2015)	78	Retrospective	EA, LA	7/43 (16)	6/35 (17)	0.919			
Swenson et al. (2018)	72	Retrospective	EA, LA	4/53 (8)	3/19 (16)	0.556			
Shohat et al. (2020)	1174	Retrospective	EA, LA	201/769 (26)	93/405 (23)	0.257			
Nurmohamed et al. (2021)	67	Retrospective	EA, LA	7/44 (16)	5/23 (22)	0.555^*^			
Zhu et al. (2021)	230	Retrospective	EA, LA, C	12/124 (10)	7/106 (7)	0.399			
Serum CRP	DAIR ( n )	Study design	Type of	Success	Failure	p value			
			infection	mean CRP	mean CRP				
				(SD)	(SD)				
Tornero et al. (2015)	222	Retrospective	EA	31 mg L^−1^ (–)	147 mg L^−1^ (–)	<0.001			
Löwik et al. (2018)	386	Retrospective	EA	79 mg L^−1^ (86)	132 mg L^−1^ (108)	<0.001			
Shohat et al. (2020)	1174	Retrospective	EA, LA	115 mg L^−1^ (115)	149 mg L^−1^ (113)	<0.001			
Zhu et al. (2021)	230	Retrospective	EA, LA, C	106 mg L^−1^ (98)	163 mg L^−1^ (127)	0.0002			
Swenson et al. (2018)	72	Retrospective	EA, LA	118 mg L^−1^ (–)	153 mg L^−1^ (00)	0.249			
Chang et al. (2022)	101	Retrospective	EA, LA	116 mg L^−1^ (85)	166 mg L^−1^ (125)	0.082			
ASA	DAIR ( n )	Study design	Type of	Failure	Failure	p value			
			infection	rate ASA	rate ASA				
				1+2	3+4				
Triantafyllopoulos et al. (2015)	78	Retrospective	EA, LA	20/49 (41)	15/29 (52)	0.349^*^			
Grammatopoulos et al. (2017a)	82	Retrospective	all PJIs	22/48 (46)	9/34 (26)	0.075^*^			
Grammatopoulos et al. (2017b)	122	Retrospective	all PJIs	31/71 (44)	18/51 (35)	0.352^*^			
Löwik et al. (2018)	386	Retrospective	EA	83/231 (36)	65/155 (42)	0.234			
Swenson et al. (2018)	72	Retrospective	EA, LA	4/19 (21)	15/53 (28)	0.845			
Wouthuyzen-Bakker et al. (2019)	340	Retrospective	LA	62/148 (42)	64/140 (46)	0.52			
Toh et al. (2021)	106	Retrospective	EA, LA	15/61 (25)	17/45 (38)	0.131			
Nurmohamed et al. (2021)	67	Retrospective	EA, LA	16/43 (37)	7/24 (29)	0.506^*^			
Zhu et al. (2021)	230	Retrospective	EA, LA, C	61/123 (50)	45/107 (42)	0.253			
Bernaus et al. (2022)	376	Retrospective	EA	13/157 (8)	31/219 (14)	0.081			

DAIR – debridement, antibiotics, and implant retention, EA – early acute infection, LA – late acute infection, C – chronic infection, NS – not significant, IT – immunosuppressive therapy. ^*^ Chi-squared test. NA – not available.

#### 3.4.1 Age

Older age has been identified as important variable associated with DAIR failure (Shohat et al., 2020; Löwik et al., 2018; Davis et al., 2022). While Shohat et al. (2020) were not able to detect a difference in their univariate analysis of 1174 patients with an early or late acute infection treated with DAIR ( p=0.432 ), random forest analysis revealed older age as one of the important factors associated with failure. In addition, Wouthuyzen-Bakker et al. (2019) found in their retrospective multicentre study of 340 patients with late acute PJIs a statistically significant higher failure rate in patients older than 80 years treated with DAIR (adjusted OR 2.6, 95 % CI: 1.15–5.91; p=0.02 ). In a retrospective study of 386 early acute PJIs, patients who failed treatment were significantly older than successful patients (mean age: 75 a ± 11 vs. 72 a ± 11, p=0.00 9) (Löwik et al., 2018). However, other authors were not able to find such a correlation between treatment failure and age (Table 7) (Triantafyllopoulos et al., 2015; Zmistowski et al., 2016; Swenson et al., 2018; Shohat et al., 2020; Zhu et al., 2021; Chang et al., 2022; Veerman et al., 2022b; Bernaus et al., 2022). Additionally, there was no clear correlation between failure and patients > 70 years in the latter mentioned study ( p=0.152 ) (Löwik et al., 2018). Interestingly, Kunutsor et al. (2018) found in their meta-analysis of 93 articles including 4897 PJIs that infection control was higher in older patients ( ≥70 years) compared to younger patients ( <70 years) ( p=0.022 ). However, this may be explained by the fact that elderly patients are less likely to undergo invasive revision surgery and, hence, may be underrepresented in the treatment failure group when treatment failure was only defined as further surgery. Suppressive antibiotic therapy was not included as failure at all. Nevertheless, there is currently no clear consensus on age, and further high-quality studies are needed on this topic. However, patients > 80 years of age certainly have a higher risk of DAIR failure, which should be considered in the decision-making process.

#### 3.4.2 Male sex

Male sex was also identified as a risk factor for failure in patients treated with DAIR (Shohat et al., 2020; Löwik et al., 2018). In a univariate analysis of 1174 patients undergoing DAIR, male patients (38 %) showed a higher failure rate in comparison to females (32 %, p=0.027 ) (Shohat et al., 2020). In a multivariate analysis of 340 late acute PJIs treated with DAIR, male sex was associated with a worse outcome (OR 2.02, 95 % CI: 1.05–3.89; p=0.04 ) (Wouthuyzen-Bakker et al., 2019). Similar results were observed in a cohort of 386 early acute PJIs (male sex) (Löwik et al., 2018). Additionally, it was reported that male patients have a higher incidence of systemic sepsis (Ludwick et al., 2022; Annane et al., 2003; Shapiro et al., 2008). Ludwick et al. (2022) demonstrated an association between male sex and development of sepsis in their study of 236 patients who underwent a DAIR procedure (OR 1.96, 95 % CI: 1.03–3.81; p=0.04 ). In contrast to the above-mentioned studies, many other authors were not able to find a correlation between male sex and DAIR failure (Table 7) (Zmistowski et al., 2016; Lora-Tamayo et al., 2017; Triantafyllopoulos et al., 2015; Swenson et al., 2018; Nurmohamed et al., 2021; Toh et al., 2021; Zhu et al., 2021; Veerman et al., 2022b; Bernaus et al., 2022; Chang et al., 2022). Hence, due to the controversies in the literature, the influence of sex as a risk factor for DAIR failure is still unclear.

#### 3.4.3 Rheumatoid arthritis

Rheumatoid arthritis (RA) was shown to be a host-related factor associated with DAIR failure (Lora-Tamayo et al., 2013, 2017; Wouthuyzen-Bakker et al., 2019; Shohat et al., 2020). Lora-Tamayo et al. (2017) demonstrated a higher risk of early failure in RA patients in their retrospective cohort study of 345 *S. aureus* PJIs (OR 3.88, 95 % CI: 1.44–10.4; p=0.007 ) (Lora-Tamayo et al., 2013) as well as in their study of 444 streptococcal PJIs treated with DAIR (OR 2.23, 95 % CI: 1.45–3.43; p<0.01 ). In late acute PJIs, an even higher risk was observed (DAIR failure: RA: 74 % vs. non-RA: 43 %, p<0.001 ) (Wouthuyzen-Bakker et al., 2019). RA was an independent predictor of DAIR failure with an OR of 3.87 (95 % CI: 1.59–9.41; p=0.001 ) in their multivariate analysis (Wouthuyzen-Bakker et al., 2019). Additionally, a clear correlation between RA and DAIR failure in a large cohort of early and late acute PJIs was described ( p=0.025 ) (Shohat et al., 2020). However, several other studies did not find such a correlation in their analysis (Table 7) (Zmistowski et al., 2016; Löwik et al., 2018; Swenson et al., 2018; Zhu et al., 2021; Veerman et al., 2022b). This could be explained by the fact that some studies only included early acute infections (Veerman et al., 2022b; Löwik et al., 2018) or by the small sample size with only a few RA patients.

#### 3.4.4 Immunosuppressive therapy

The use of immunosuppressive therapy was also associated with a worse outcome after DAIR (Lora-Tamayo et al., 2013, 2017; Wouthuyzen-Bakker et al., 2019; Shohat et al., 2020; Veerman et al., 2022b). In a univariate analysis, significantly higher failure rates were reported when patients were treated with immunosuppressive agents (Table 7) (Lora-Tamayo et al., 2017; Wouthuyzen-Bakker et al., 2019; Shohat et al., 2020; Veerman et al., 2022b). In a retrospective study of 444 PJI cases (early and late acute), the failure rate in patients with immunosuppressive therapy was 60 % compared to 40 % in patients without therapy ( p=0.007 ) (Lora-Tamayo et al., 2017). Similar results were reported when only late acute PJIs were investigated (immunosuppressive therapy – failure rate: 62 % vs. 43 %; p=0.03 ) (Wouthuyzen-Bakker et al., 2019) as well as only early acute PJIs (75 % vs. 28 %; p=0.012 ) (Veerman et al., 2022b). Nevertheless, some authors could not find a correlation between immunosuppressive therapy and DAIR failure (Swenson et al., 2018; Zhu et al., 2021). The small number of patients treated with immunosuppressive agents in their studies may have influenced their outcome. However, due to the inability to clear the infection, a compromised immune system may decrease the success rate after a DAIR procedure. Hence, this variable should always be considered by the treating multidisciplinary team when planning further therapy and surgery.

#### 3.4.5 Obesity and Body mass index (BMI)

While obesity and an elevated body mass index (BMI) has an increased infection risk after primary arthroplasty (Everhart et al., 2013), the risk of failure after DAIR is less clear. Urish et al. (2018) reported that BMI was one of the factors predicting failure in their study of 216 PJIs after a primary TKA following DAIR. Cox proportional hazard regression analysis revealed BMI as one of the significant univariate predictors of failure (HR 1.024, 95 % CI: 1.004–1.046; p=0.02 ) (Urish et al., 2018). Additionally, morbidly obese patients (BMI > 40 kg m^−2^) had a lower survivorship rate (40 %) in comparison to non-obese patients (BMI < 30 kg m^−2^) in a study of 114 PJI cases treated with DAIR (Katakam et al., 2020a). However, in a large cohort study including only early acute PJIs, a BMI of >35 kg m^−2^ showed no difference regarding failure rate compared to patients with a BMI of <35 kg m^−2^ ( p=0.269 ) (Löwik et al., 2018). Similar results were observed in late acute PJIs (Wouthuyzen-Bakker et al., 2019). A BMI > 30 kg m^−2^ showed no association with DAIR failure ( p=0.43 ) (Wouthuyzen-Bakker et al., 2019). In addition, when using BMI as a continuous variable, no association between BMI and DAIR failure was observed in multiple studies (Table 7) (Triantafyllopoulos et al., 2015; Zmistowski et al., 2016; Swenson et al., 2018; Shohat et al., 2020; Zhu et al., 2021; Veerman et al., 2022b; Chang et al., 2022). Interestingly, when a second DAIR was required, a BMI > 30 kg m^−2^ showed a higher success rate compared to patients with a BMI < 30 kg m^−2^ in a study by Wouthuyzen-Bakker et al. (2020a). A possible explanation may be selection bias. In their study, obese patients were younger, less often diagnosed with RA, and showed an initially lower concentration of inflammatory markers. Hence, it should not be seen as a protective variable per se. Overall, based on the current literature, the precise effect of BMI or obesity on DAIR remains unclear.

#### 3.4.6 Diabetes mellitus

Inconsistent results were reported for diabetes mellitus and DAIR (Triantafyllopoulos et al., 2015; Shohat et al., 2020). Shohat et al. (2020) showed a trend towards a higher failure rate in patients with diabetes in their retrospective study of 1174 patients with acute PJIs managed with a DAIR procedure (39 % vs. 33 %, p=0.08 ). Interestingly, in a small retrospectively analysed cohort of 78 acute PJIs treated with DAIR, a trend towards a higher success rate in patients with diabetes was observed (75 % vs. 50 %, p=0.073 ) (Triantafyllopoulos et al., 2015). A potential explanation for this controversial result may be that this study is underpowered due to the small sample size. However, in multiple other studies, no correlation between diabetes and DAIR failure or success was seen (Table 7) (Zmistowski et al., 2016; Lora-Tamayo et al., 2017; Löwik et al., 2018; Swenson et al., 2018; Wouthuyzen-Bakker et al., 2019; Toh et al., 2021; Zhu et al., 2021; Veerman et al., 2022b; Chang et al., 2022; Urish et al., 2018). Hence, the impact of diabetes mellitus on DAIR is also not completely clear. However, due to the available literature it seems that patients with diabetes do not have a worse outcome in comparison to patients without this comorbidity (especially if well controlled).

#### 3.4.7 Chronic renal failure

Chronic renal failure was also identified as an independent predictor of DAIR failure in some studies (Lora-Tamayo et al., 2017; Tornero et al., 2015; Bernaus et al., 2022). When patients with chronic renal failure were treated with DAIR, an odds ratio of 5.92 (95 % CI 1.47–23.85) was observed in a retrospective study of 222 early acute PJI cases (Tornero et al., 2015). Bernaus et al. (2022) also demonstrated a higher failure rate after DAIR in patients with early acute PJI and chronic renal failure (21 % vs. 10 %, p=0.045 ). However, in late acute infections, this phenomenon was not seen. Individuals with chronic renal failure showed no increased risk to fail in studies including late acute infections (Table 7) (Triantafyllopoulos et al., 2015; Lora-Tamayo et al., 2017; Wouthuyzen-Bakker et al., 2019; Shohat et al., 2020; Zhu et al., 2021). Hence, it seems that only patients with an early acute infection and chronic renal failure are at higher risk of DAIR failure. Nevertheless, Löwik et al. (2018) were not able to find an association between chronic renal failure and DAIR failure in their study only investigating early acute PJIs (failure rate with chronic renal failure: 42 %, without: 38 %, p=0.667 ). Due to these inconsistent results, there is currently no clear consensus on the impact of chronic renal failure on the outcome of DAIR.

#### 3.4.8 Liver cirrhosis

Liver cirrhosis was also associated with worse outcomes in a retrospectively conducted study of 222 early acute PJIs, showing a higher failure rate in patients with this comorbidity (48 % vs. 21 %, p=0.004 ). However, other authors were not able to find a correlation between cirrhosis and DAIR failure (Table 7) (Löwik et al., 2018; Triantafyllopoulos et al., 2015; Wouthuyzen-Bakker et al., 2019; Bernaus et al., 2022). An explanation for these results may be the small number of patients with liver disease included in these latter studies (Table 7). Nevertheless, in a large cohort of 1174 acute PJIs, 41 patients (3 %) had liver cirrhosis, and almost half failed after DAIR (49 %) (Shohat et al., 2020). Although not at a statistically significant level, a trend towards a higher failure rate in these patients was demonstrated (49 % vs. 34 % p=0.065 ). Further studies including a higher number of these patients are needed to elucidate the effect of liver cirrhosis on DAIR outcomes.

#### 3.4.9 Nicotine use and chronic obstructive pulmonary disease (COPD)

While nicotine use (smoking) showed no significant correlation with DAIR failure in the literature (Triantafyllopoulos et al., 2015; Swenson et al., 2018; Shohat et al., 2020; Nurmohamed et al., 2021; Zhu et al., 2021), chronic obstructive pulmonary disease (COPD) did (Shohat et al., 2020). In a large cohort study of early acute ( n=790 ) and late acute PJIs ( n=338 ) managed with DAIR, the failure rate in patients with COPD was 41 % compared to 33 % in patients without COPD ( p=0.048 ) (Shohat et al., 2020). However, it was not 1 of the 10 most important factors associated with failure in their random forest analysis (Shohat et al., 2020). In another large study of late acute PJIs, multivariate analysis revealed COPD as a significant independent predictor of early DAIR failure (OR 4.26; 95 % CI: 1.62–11.17; p=0.003 ) (Wouthuyzen-Bakker et al., 2019). In addition, in a study only including early acute PJIs, a higher failure rate in COPD patients was observed (47 % vs. 36 %) but not at a statistically significant level ( p=0.074 ). However, Zhu et al. (2021) did not find an association between COPD and DAIR failure in their study including 230 patients, but again the number of patients with COPD was small in their cohort ( n=9 , Table 7). Nevertheless, due to the available data, it seems that COPD may have an impact on DAIR failure in early as well as late acute PJI cases.

#### 3.4.10 C-reactive protein (CRP)

Serum C-reactive protein (CRP) at clinical presentation is one of the most studied clinical factors associated with DAIR failure (Table 7) (Löwik et al., 2018; Shohat et al., 2020; Zhu et al., 2021; Bernaus et al., 2022; Tornero et al., 2015; Ludwick et al., 2022). Three large cohort studies of early acute PJIs showed that patients with a preoperative CRP level > 11.5 mg dL^−1^ had a higher failure rate following DAIR compared to patients with a lower CRP concentration (Bernaus et al., 2022: 18 % vs. 9 %, p= 0.009; Löwik et al., 2018: 55 % vs. 30 %, p<0.001 ; Tornero et al., 2015: 56 % vs. 12 %, p<0.001 ). In late acute PJIs, a CRP > 11.5 mg dL^−1^ was shown to be an independent predictor of early failure (Wouthuyzen-Bakker et al., 2019). When using CRP as a continuous variable, higher CRP levels were observed in failed cases (Table 7) (Löwik et al., 2018; Shohat et al., 2020; Zhu et al., 2021; Chang et al., 2022). Additionally, a second DAIR needed to be performed more often in cases with a CRP > 11.5 mg dL^−1^ at initial presentation (45 % vs. 34 %, p=0.03 ) (Wouthuyzen-Bakker et al., 2020a). Hence, it appears that a high preoperative serum CRP level at clinical presentation may be a surrogate marker for the severity of the infection and may predict DAIR success or failure.

The effect of other host and clinical factors (e.g. heart failure, Tornero et al., 2015; thyroid disease, Triantafyllopoulos et al., 2015; albumin, Davis et al., 2022, erythrocyte sedimentation rate (ESR), Toh et al., 2021; haematocrit (HCT), Swenson et al., 2018) on DAIR failure is currently not clear in the available literature.

#### 3.4.11 General classification systems

While one single host or clinical factor does not seem to be an absolute contraindication for a DAIR procedure, classification systems or scores based on a combination of different comorbidities and clinical factors can be used to decide which patient may profit from it (American Association of Anesthesiologists (ASA) score, Charlson Comorbidity Index (CCI), McPherson classification). Indeed, a correlation between higher ASA scores and DAIR failure was described in the literature (Fink et al., 2017). Fink et al. (2017) showed that the risk of failure increased 7-fold with an increasing ASA classification in a retrospective study of 67 acute infections (early acute: n=39 , late acute: n=28 ). Löwik et al. (2018) observed a significantly higher mean ASA score in patients who failed after DAIR ( 2.44±0.60 ) in comparison to successful cases ( 2.29±0.65 , p=0.021 ), but patients with an ASA score ≥3 showed no higher failure rate (ASA 1+2 : 36 %, ASA 3+4 : 42 %; p=0.234 ; Table 7). Regarding DAIR failure, no difference was seen in multiple studies between patients with an ASA score of 1 or 2 and patients with a score of 3 or 4 (Table 7) (Triantafyllopoulos et al., 2015; Grammatopoulos et al., 2017b; Swenson et al., 2018; Wouthuyzen-Bakker et al., 2019; Toh et al., 2021; Nurmohamed et al., 2021; Zhu et al., 2021). Interestingly, Grammatopoulos et al. (2017a) described a trend towards a higher failure rate in patients with a lower ASA score (ASA 1+2 : 46 %, ASA 3+4 : 26 %) but not at a significant level ( p=0.075 ). However, their sample size was small ( n=78 ), and a detailed definition of failure (e.g. antimicrobial suppressive therapy) was not provided in their study (Grammatopoulos et al., 2017a).

Amongst others, Bryan et al. (2017) investigated the DAIR failure rate in 90 acute hip infections based on the McPherson host grading. A higher host grade was associated with a higher failure rate: patients with a host grade A showed a failure rate of 8 %, in patients with a host grade B, it was 16 % (HR 3.4, 95 % CI: 1.2–11.0, p=0.04 ), and in patients with a host grade C 44 % (HR 7.6, 95 % CI: 1.5–14.6, p=0.006 ) (Bryan et al., 2017).

In general, it appears that a fitter and healthier host may have greater chances of DAIR success and that the combination of comorbidities is more important than one factor alone. This should be considered in the preoperative decision-making process of the multidisciplinary team.

#### 3.4.12 Preoperative risk scores (KLIC score, CRIME-80 score) and machine learning

Preoperative risk scores more tailored to acute PJIs were developed to help treating surgeons and physicians selecting patients most eligible for a DAIR procedure (Tornero et al., 2015; Wouthuyzen-Bakker et al., 2019).

In early acute PJIs, the KLIC score was introduced to preoperatively identify patients who would benefit from DAIR or are at high risk of DAIR failure (Tornero et al., 2015). Tornero et al. (2015) identified five preoperative factors as independent predictors of failure in their retrospective study including 222 early acute PJIs: chronic renal failure (kidney, OR 5.92, 95 % CI: 1.47–23.85), liver cirrhosis (OR 4.46, 95 % CI: 1.15–17.24), revision surgery (OR 4.34, 95 % CI: 1.34–14.04), or femoral neck fracture (OR 4.39, 95 % CI: 1.16–16.62) compared with primary arthroplasty, CRP > 11.5 mg dL^−1^ (OR 12.308, 95 % CI: 4.56–33.19), and cemented prosthesis (OR 8.71, 95 % CI: 1.95–38.97). With these factors, an additive scoring tool predicting DAIR failure was designed by using logistic regression analysis. The failure rates of scores ≤ 2 (group A), >2 –3.5 (group B), 4–5 (group C), >5 –6.5 (group D), and ≥7 (group E) were 4.5 %, 19.4 %, 55 %, 71.4 %, and 100 %, respectively. This score was highly predictive of early failure (at 60 d post-surgery) in their study. However, in a large external validation study of 455 patients with early acute PJIs, failure rates were 12 % for group A, 18 % for group B, 26 % for group C, 24 % for group D, and 0 % for group E (Bernaus et al., 2022). In this cohort, no difference in failure was seen between consecutive groups before 60 d of the DAIR procedure in a univariable analysis. After 60 d, only groups B (4 %) and C (20 %) showed a difference in DAIR failure in a univariable analysis ( p=0.006 ). The authors demonstrated an increased failure risk for groups A, B, and C but a decreased tendency for groups D and E. Hence, the KLIC score was not able to predict failure in their cohort (Bernaus et al., 2022). In another study by Chalmers et al. (2021), the AUCs of KLIC, CCI, and McPherson host grade were all below 0.66 at 90 d as well as at 2 years following DAIR. The authors concluded that alternative scores for predicting DAIR outcomes are needed (Chalmers et al., 2021). Three other external studies also validated the KLIC score showing a predictive power only in cases with a very low or high score; average scores were less useful (Löwik et al., 2018; Dx Duffy et al., 2018; Jiménez-Garrido et al., 2018). Additionally, other factors were found to be more predictive of failure in some of the above-mentioned studies (Löwik et al., 2018; Bernaus et al., 2022).

In late acute PJIs, a similar analysis was done by Wouthuyzen-Bakker et al. (2019) to create a preoperative scoring tool to identify patients most eligible for DAIR. COPD, CRP > 150 mg L^−1^, rheumatoid arthritis, indication for the prosthesis (e.g. fracture), male sex, not being able to exchange mobile parts, and age above 80 years were identified as the strongest variables for failure in their retrospective study including 340 late acute PJIs (Wouthuyzen-Bakker et al., 2019). Scores of -1 , 0, 1–2, 3–4, and ≥5 showed failure rates of 22 %, 28 %, 40 %, 64 %, and 79 %, respectively. When PJI was caused by *S. aureus*, higher failure rates were observed (43 %, 42 %, 45 %, 77 %, 100 %). Indeed, detection of *S. aureus* was one of the major predictors of DAIR failure in their cohort. Additionally, in these cases, the other factors were less predictive of failure. Hence, the authors highlighted the importance of isolating the causative microorganism prior to surgery. They concluded that the CRIME-80 score may be useful in identifying high-risk patients especially with infections caused by another microorganism than *S. aureus* (Wouthuyzen-Bakker et al., 2019).

However, Chalmers et al. (2021) only observed an AUC of the CRIME-80 score of 0.7 (95 % CI: 0.557–0.838) at 90 d and 0.613 (95 % CI: 0.499–0.726) at 2 years in their cohort of 134 late acute infections. Hence, they concluded that alternative scores for predicting DAIR in late acute PJIs are needed (Chalmers et al., 2021).

In 2020, Shohat et al. (2020) used machine learning to develop a tool for predicting DAIR outcomes and validated it in their large retrospective multi-centre study of 1174 patients. Serum CRP, positive blood cultures, indication for index arthroplasty other than osteoarthritis, not exchanging the modular components, immunosuppressive therapy, late acute infections, MRSA infection, overlying skin infection, polymicrobial infection, and older age were the 10 most important factors using random forest analysis (ordered by importance). Their created model had an AUC of 0.74, showing a good discriminatory capability (Shohat et al., 2020). In their cross-validation, similar probabilities between predicted and observed failures were seen, which indicates the high accuracy of this algorithm. However, to the best of our knowledge, no external validation study has yet been conducted. Nevertheless, this algorithm may have great potential as an easy-to-use tool (software, smart phone app) in the future. With the ability to learn from data input, it may also help us to gain more knowledge about the important risk factors for DAIR failure and predictors of DAIR success in the future.

Preoperative risk scores including the most important host and clinical factors associated with DAIR failure and success are needed to predict the individual outcome of a DAIR procedure and, hence, help the treating team in their decision making preoperatively. Although currently available scores and algorithms show good capabilities to predict DAIR outcome and can be recommended for use in clinical practice, their AUCs are still low ( ≤0.74 ). Hence, further studies are needed to find more precise tools for predicting DAIR failure (or success) and help the treating team to find the optimal treatment option preoperatively.

#### Recommendation

Taken all together, a fit and healthy non-immunocompromised host shows the best outcome, and unhealthier patients need to be optimized (if possible) to maximize the chances of DAIR success. Based on the current literature, patients with RA, COPD, and immunosuppressive therapy seem to be associated with DAIR failure.

### 3.5 Microorganisms

The causative microorganism appeared to have an important effect on DAIR success (Qu et al., 2019; Davis et al., 2022; Wouthuyzen-Bakker et al., 2019, 2020b; Shohat et al., 2020; Chang et al., 2022; Bernaus et al., 2022; Byren et al., 2009; Toh et al., 2021; Zhu et al., 2021; Urish et al., 2018).

#### 3.5.1 Staphylococcus aureus

In multiple studies, *S. aureus* PJIs were associated with increased risk of failure and worse outcomes in comparison to infections caused by other organisms (Wouthuyzen-Bakker et al., 2019; Byren et al., 2009; Löwik et al., 2018; Swenson et al., 2018; Shohat et al., 2020; Katakam et al., 2020b; Zhu et al., 2021; Zmistowski et al., 2016; Davis et al., 2022). Overall, treatment failure rates ranged between 25 % and 56 % in more recently published studies (Table 8) (Bernaus et al., 2022; Zhu et al., 2021). In a large cohort of early and late acute infections ( n=1.174 ), an 11 % higher failure rate was reported when the infection was caused by *S. aureus* (41 % vs. 30 %, p<0.0001 ) (Shohat et al., 2020). In another study of only late acute infections treated with DAIR, *S. aureus* PJIs showed again an increased risk of failure with an OR of 3.52 (95 % CI: 1.78–6.96, p<0.001 ), especially when mobile parts were not exchanged (47.1 % vs. 36.6 %) (Wouthuyzen-Bakker et al., 2019). Similar results were observed in early acute PJIs (OR 3.27, 95 % CI: 1.55–6.89, p=0.002 ) (Bernaus et al., 2022). Interestingly, Wouthuyzen-Bakker et al. (2020b) demonstrated that the lower success rate in late acute compared to early acute infections was only seen in staphylococcal PJIs (*S. aureus*: 34 % vs. 75 %, OR 5.8, 95 % CI: 2.9–11.9, p<0.001 ; coagulase-negative staphylococci (CoNS): 46 % vs. 88 %, OR 8.8, 95 % CI: 1.4–54.8, p=0.013 ). They explained the higher failure rate in late acute *Staphylococcus* spp. infections with a possible continuous spread of bacteria originating from a distant infection source to the prosthetic joint and a higher bacterial inoculum in those cases (Wouthuyzen-Bakker et al., 2020b). Even lower success rates were reported in methicillin-resistant *S. aureus* (MRSA) PJIs compared to methicillin-susceptible *S. aureus* (MSSA) infections (57 % vs. 63 %, p<0.0001 ) (Shohat et al., 2020). The presence of MRSA was 1 of the 10 most important predictors of failure in their study (Shohat et al., 2020). In another study of only *S. aureus* infections ( n=345 ), no prognostic differences between MRSA and MSSA PJIs were seen, but differences regarding time to failure were observed (Lora-Tamayo et al., 2013). A large proportion of MRSA infections (88 %) failed very early during the first week (on antimicrobial therapy), while MSSA PJIs frequently failed once antimicrobial therapy was withdrawn (Lora-Tamayo et al., 2013). Nevertheless, due to the high failure rate, some authors recommended the identification of the causing microorganism(s) preoperatively and a more invasive treatment strategy (exchange of implant, one-stage or two-stage revision) when *S. aureus* is present (Tarity et al., 2021; Wouthuyzen-Bakker et al., 2019, 2021; Toh et al., 2021; Zhu et al., 2021), especially in late acute infections (Wouthuyzen-Bakker et al., 2020b). However, other (smaller) studies did not find a lower success rate in *S. aureus* infections treated with DAIR (Grammatopoulos et al., 2017b; Flierl et al., 2017; Ottesen et al., 2019).

**Table 8:** A comparison of the literature of DAIR failure rates (FRs) based on the causing microorganism.

*Staphylococcus aureus*	DAIR ( n )	Study design	Type of	MO	FR	FR	p value	FR other MO w/o	p value
			infection		*S. aureus*	other MO		polymicrobial	
Byren et al. (2009)	112	Retrospective	All PJIs	*S. aureus*	13/47 (28)	5/26 (19)	0.573	NA	NA
Zmistowski et al. (2016)	153	Retrospective	NA	MSSA + MRSA	35/65 (55)	38/88 (43)	0.252	30/70 (43)	0.230
Grammatopoulos et al. (2017b)	122	Retrospective	All PJIs	*S. aureus*	12/39 (31)	26/80 (33)	1.000	11/45 (24)	0.625
Löwik et al. (2018)	386	Retrospective	EA	*S. aureus*	86/181 (48)	62/205 (30)	0.001	NA	NA
Swenson et al. (2018)	72	Retrospective	EA, LA	*S. aureus*	14/29 (48)	5/43 (12)	0.001	NA	NA
Wouthuyzen-Bakker et al. (2019)	340	Retrospective	LA	*Staph. aureus*	76/139 (55)	74/264 (28)	<0.0001	NA	NA
Shohat et al. (2020)	1174	Retrospective	EA, LA	MSSA	80/215 (37)	325/959 (34)	0.383	197/630 (31)	0.111
				MRSA	128/298 (43)	277/876 (32)	0.001	149/547 (27)	<0.0001
				MSSA + MRSA	208/513 (41)	197/661 (30)	<0.0001	69/332 (21)	<0.0001
Katakam et al. (2020a)	263	Retrospective	NA	MSSA, MRSA	61/118 (52)	49/145 (34)	0.004	NA	NA
Zhu et al. (2021)	230	Retrospective	EA, LA, C	*S. aureus*	45/81 (56)	61/149 (41)	0.038	40/100 (40)	0.051
Chang et al. (2022)	67	Retrospective	LA	MSSA, MRSA, MRSE	9/24 (38)	10/43 (23)	0.263	7/40 (18)	0.134
Bernaus et al. (2022)	455	Retrospective	EA	MSSA, MRSA	32/128 (25)	35/177 (20)	0.327	15/101 (15)	0.070
CoNS	DAIR ( n )	Study design	Type of	MO	FR	FR	p value	FR other MO	p value
			infection		CoNS	other MO		(w/o *S. aureus*)	
Zmistowski et al. (2016)	153	Retrospective	NA	CoNS	7/16 (44)	66/137 (48)	0.796	31/71 (44)	1.000
Grammatopoulos et al. (2017b)	122	Retrospective	All PJIs	CoNS	7/22 (32)	31/97 (32)	1.000	19/58 (33)	1.000
Löwik et al. (2018)	386	Retrospective	EA	CoNS	41/126 (33)	107/260 (41)	0.118	21/79 (27)	0.435
Swenson et al. (2018)	72	Retrospective	EA, LA	CoNS	3/11 (27)	16/61 (26)	1.000	2/32 (6)	0.096
Wouthuyzen-Bakker et al. (2019)	340	Retrospective	LA	CoNS	12/30 (40)	138/297 (46)	0.567	62/158 (39)	1.000
Shohat et al. (2020)	1174	Retrospective	EA, LA	*S. epidermidis*	91/285 (32)	314/889 (35)	0.316	106/376 (28)	0.304
Zhu et al. (2021)	230	Retrospective	EA, LA, C	CoNS	20/51 (39)	86/179 (48)	0.339	41/98 (42)	0.861
Streptococci	DAIR ( n )	Study design	Type of	MO	FR	FR	p value	FR other MO	p value
			infection		streptococci	other MO		(w/o *S. aureus*)	
Zmistowski et al. (2016)	153	Retrospective	NA	Streptococci	4/11 (36)	69/142 (49)	0.539	34/77 (44)	0.751
Grammatopoulos et al. (2017b)	122	Retrospective	All PJIs	Streptococci	1/11 (9)	37/108 (34)	0.171	25/69 (36)	0.093
Löwik et al. (2018)	386	Retrospective	EA	Streptococci	22/66 (33)	126/320 (39)	0.405	40/143 (28)	0.515
Swenson et al. (2018)	72	Retrospective	EA, LA	Streptococci	2/18 (11)	17/54 (31)	0.125	3/25 (12)	1.000
Wouthuyzen-Bakker et al. (2019)	340	Retrospective	LA	Streptococci	36/97 (37)	114/230 (50)	0.040	38/91 (42)	0.552
Katakam et al. (2020a)	263	Retrospective	NA	Streptococci	15/42 (36)	95/221 (43)	0.400	34/103 (33)	0.847
Shohat et al. (2020)	1174	Retrospective	EA, LA	Streptococci	66/194 (34)	339/980 (35)	0.934	131/467 (28)	0.136
Zhu et al. (2021)	230	Retrospective	EA, LA, C	Streptococci	27/52 (52)	79/178 (44)	0.348	34/97 (35)	0.055
Chang et al. (2022)	67	Retrospective	LA	Streptococci	2/15 (14)	17/52 (33)	0.200	8/28 (29)	0.451

**Table 8:** Continued.

Enterococci	DAIR ( n )	Study design	Type of	MO	FR	FR	p value	FR other MO	p value
			infection		Enterococci	other MO		(w/o *S. aureus*)	
Zmistowski et al. (2016)	153	Retrospective	NA	Enterococci	1/3 (33)	72/150 (48)	1.000	37/85 (44)	1.000
Löwik et al. (2018)	386	Retrospective	EA	Enterococci	26/70 (37)	122/316 (39)	0.892	36/135 (27)	0.149
Wouthuyzen-Bakker et al. (2019)	340	Retrospective	LA	Enterococci	8/11 (73)	142/316 (45)	0.120	66/177 (37)	0.026
Shohat et al. (2020)	1174	Retrospective	EA, LA	Enterococci	45/128 (35)	360/1046 (34)	0.922	152/533 (29)	0.162
Bernaus et al. (2022)	455	retrospective	EA	Enterococci	2/24 (8)	65/281 (23)	0.123	33/153 (22)	0.172
Gram	DAIR ( n )	Study design	Type of	MO	FR	FR	p value	FR other MO	p value
negatives			infection		Gram negatives	other MO		(w/o *S. aureus*)	
Zmistowski et al. (2016)	153	Retrospective	NA	Gram negatives	5/9 (56)	68/144 (47)	0.737	33/79 (42)	0.492
Grammatopoulos et al. (2017b)	122	Retrospective	All PJIs	Gram negatives	3/12 (25)	35/107 (33)	0.750	23/68 (34)	0.742
Löwik et al. (2018)	386	Retrospective	EA	Gram negatives	32/75 (43)	116/311 (37)	0.428	30/130 (23)	0.005
Wouthuyzen-Bakker et al. (2019)	340	Retrospective	LA	Gram negatives	18/50 (36)	132/277 (48)	0.165	56/138 (41)	0.615
Shohat et al. (2020)	1174	Retrospective	EA, LA	Gram negatives	77/210 (37)	328/887 (37)	1.000	120/374 (32)	0.275
Zhu et al. (2021)	230	Retrospective	EA, LA, C	Gram negatives	19/39 (49)	87/191 (46)	0.728	42/110 (38)	0.262
Polymicrobial	DAIR ( n )	Study design	Type of	MO	FR	FR	p value	FR other MO	p value
infections			infection		polymicrobial	other MO		(w/o *S. aureus*)	
Zmistowski et al. (2016)	153	Retrospective	NA	Polymicrobial	8/18 (44)	65/135 (48)	0.807	30/70 (43)	1.000
Löwik et al. (2018)	386	Retrospective	EA	Polymicrobial	67/176 (38)	81/210 (39)	1.000	NA	NA
Swenson et al. (2018)	72	Retrospective	EA, LA	Polymicrobial	4/16 (25)	15/44 (34)	0.754	NA	NA
Shohat et al. (2020)	1174	Retrospective	EA, LA	Polymicrobial	128/329 (39)	277/845 (33)	0.056	69/332 (21)	<0.0001
Zhu et al. (2021)	230	Retrospective	EA, LA, C	Polymicrobial	21/49 (43)	85/181 (47)	0.632	40/100 (40)	0.859
Bernaus et al. (2022)	455	Retrospective	EA	Polymicrobial	20/76 (26)	47/229 (21)	0.337	15/101 (15)	0.085
Chang et al. (2022)	67	Retrospective	LA	Polymicrobial	3/3 (100)	16/64 (25)	0.020	7/40 (18)	0.01
Culture	DAIR ( n )	Study design	Type of	MO	FR	FR	p value	FR culture +	p value
negatives			infection		culture	culture		(w/o *S. aureus*)	
					negative	positives			
Zmistowski et al. (2016)	153	Retrospective	NA	Culture negatives	12/28 (43)	61/125 (49)	0.677	26/60 (43)	1.000
Swenson et al. (2018)	72	Retrospective	EA, LA	Culture negatives	0/12 (0)	19/60 (32)	0.028	5/31 (16)	0.300
Löwik et al. (2018)	386	Retrospective	EA	Culture negatives	22/84 (26)	126/302 (42)	0.011	40/121 (33)	0.354
Katakam et al. (2020a)	263	Retrospective	NA	Culture negatives	14/60 (23)	96/203 (47)	0.001	35/85 (41)	0.032
Tirumala et al. (2021)	149	Retrospective	EA, LA	Culture negatives	6/46 (13)	20/103 (19)	0.484	NA	NA
Chang et al. (2022)	67	Retrospective	LA	Culture negatives	2/14 (14)	17/53 (32)	0.318	8/29 (28)	0.456

DAIR – debridement, antimicrobial therapy, and implant retention, EA – early acute infection, LA – acute haematogenous infection, C – chronic infection, NS – not significant, MO – microorganism, NA – not available, CoNS – coagulase-negative staphylococci, MSSA – methicillin-susceptible *S. aureus*, MRSA – methicillin-resistant *S. aureus*, MRSE – methicillin-resistant *S. epidermidis*. For comparison analysis, Fisher's exact test was used.

The failure rates of other common microorganisms varied enormously in the literature (Table 8): 9 %–52 % for streptococci (Grammatopoulos et al., 2017b; Zhu et al., 2021), 27 %–44 % for coagulase negative staphylococci (CoNS) (Swenson et al., 2018; Zmistowski et al., 2016), 8 %–73 % for enterococci (Zmistowski et al., 2016; Wouthuyzen-Bakker et al., 2019), and 25 %–56 % for Gram-negative bacteria (Zmistowski et al., 2016; Grammatopoulos et al., 2017b).

#### 3.5.2 *Streptococcus* spp.

In streptococcal PJIs, better outcomes were seen in multiple studies (Table 8) (Grammatopoulos et al., 2017b; Wouthuyzen-Bakker et al., 2019; Swenson et al., 2018). Grammatopoulos et al. (2017b) described a lower complication and reoperation rate after a DAIR procedure in PJIs caused by *Streptococcus* spp. (9 % vs. 34 %). Similar results were seen in late acute infections (37 % vs. 50 %, p=0.039 ) (Wouthuyzen-Bakker et al., 2019). Although not at a statistically significant level, most recently published studies showed more favourable outcomes in streptococcal PJI compared to PJIs caused by other pathogens (Table 8) (Zmistowski et al., 2013; Löwik et al., 2018; Katakam et al., 2020b; Shohat et al., 2020; Zhu et al., 2021; Chang et al., 2022).

#### 3.5.3 *Enterococcus* spp.

Outcomes of enterococcal infections were infrequently reported. In a large cohort of only late acute PJIs, infections had the highest failure rate (73 %, *Enterococcus faecium* 80 %, *E. faecalis* 67 %, p=0.069 ), but the sample size was small ( n=11 , *E. faecium*: 5, *E. faecalis*: 6) (Wouthuyzen-Bakker et al., 2019). In contrast, a very low DAIR failure rate of 8 % was observed in a study of only early acute infections (8 % vs. non-enterococcal PJI: 23 %, p=0.093 ) (Bernaus et al., 2022). However, the number of enterococcal PJIs was again limited ( n=24 ) (Bernaus et al., 2022). The difference between these studies may be explained by the type of infection (early vs. late acute) and limited sample size. Nevertheless, other authors were not able to calculate a higher risk of failure in enterococcal PJIs (Table 8) (Zmistowski et al., 2016; Löwik et al., 2018; Shohat et al., 2020). Due to this inconsistency in the literature, no conclusion can be drawn at the moment.

#### 3.5.4 CoNS and Gram-negative bacteria

In univariate analyses, the presence of CoNS and Gram-negative bacteria did not seem to influence DAIR outcomes (Table 8) (Zmistowski et al., 2016; Grammatopoulos et al., 2017b; Löwik et al., 2018; Swenson et al., 2018; Wouthuyzen-Bakker et al., 2019, 2020b; Shohat et al., 2020; Zhu et al., 2021).

#### 3.5.5 Polymicrobial infections

In some studies, polymicrobial infections were associated with a higher risk of DAIR failure (Bernaus et al., 2022; Ludwick et al., 2022; Chang et al., 2022). A 100 % failure rate was observed in a cohort of 67 late acute PJIs. However, multiple organisms were only identified in three PJI cases ( n=3 /67) (Chang et al., 2022). Shohat et al. (2020) showed that 39 % ( n=128 /329) of polymicrobial PJIs failed in comparison to 33 % ( n=277 /845) of monomicrobial infections ( p=0.056 ). Additionally, Ludwick et al. (2022) reported the highest reinfection risk in septic patients with MRSA and polymicrobial infections. The worse outcome in these cases may be explained by the presence of *S. aureus* amongst other organisms. However, no detailed description of the causing microorganisms in polymicrobial infections is available in their studies. Nevertheless, other authors were not able to show a statistically significant difference between poly- and monomicrobial PJIs (Table 8) (Zhu et al., 2021; Swenson et al., 2018).

#### 3.5.6 Culture-negative infections

Cultures can be negative in up to 46 % of PJIs depending on the used infection definition (Sousa et al., 2023). These culture-negative infections showed better outcomes compared to culture-positive cases following DAIR in the literature (Swenson et al., 2018; Löwik et al., 2018; Katakam et al., 2020b; Zmistowski et al., 2013; Tirumala et al., 2021; Chang et al., 2022). All studies listed in Table 8 showed a lower failure rate in culture-negative compared to culture-positive PJIs. In three of these studies, a statistically significant difference between both groups was observed ( p<0.05 ) (Swenson et al., 2018; Löwik et al., 2018; Katakam et al., 2020b). Interestingly, Urish et al. (2018) found a higher failure risk when no pathogen could be identified (Urish et al., 2018). However, the included number of patients was small in their study (28 culture-negative cases of 153 PJI patients). Overall, it seems that culture-negative PJIs have a better outcome compared to culture-positive cases.

It needs to be emphasized that different antimicrobial regimens can influence DAIR outcomes, and, hence, data on microorganisms need to be interpreted with caution.

#### Recommendation

Due to the available results in the literature, it would be beneficial to identify the causing microorganism and its susceptibility pattern prior to surgery. It should be considered when a decision for further surgical and antimicrobial treatment is made. However, further management (DAIR) should not be delayed in all cases until microbiological results are available as a symptom onset of >3 weeks can reduce the success rate. In acute infections (especially late acute PJIs) caused by *S. aureus* (MSSA and MRSA), an individual multidisciplinary team discussion considering other host and clinical factors is important to find the optimal treatment strategy for these patients.

### 3.6 Bacteraemia/sepsis

In the literature, bacteraemia is one of the most important factors for DAIR failure (Table 9) (Lora-Tamayo et al., 2013, 2017; Löwik et al., 2018; Ludwick et al., 2022; Wouthuyzen-Bakker et al., 2019). If bacteraemia or sepsis is present, a higher failure rate was demonstrated ranging between 48 % and 65 % (Table 9). Shohat et al. (2020) analysed the 10 most important risk factors for failure of DAIR in their multicentre retrospective study. In their univariate analysis, a positive blood culture was a risk factor for failure ( p<0.001 ). Wouthuyzen-Bakker et al. (2019) observed a 15 % higher failure rate in blood-culture-positive PJIs ( n=61 /109, 56 %) compared to blood-culture-negative cases ( n=62 /150, 41 %) in their multicentre retrospective observational study of 340 late acute infections ( p=0.02 ). It was highlighted that a continuous spread of microorganisms to the prosthetic joint in blood-culture-positive cases may be the possible reason for the high failure rate in these patients. Additionally, in their study, bacteraemia was more common in patients with fever ( p=0.007 ), hip PJIs ( p=0.001 ), endocarditis ( p=0.001 ), *S. aureus* infections ( p<0.001 ), and implants of >2 years of age ( p=0.003 ) (Wouthuyzen-Bakker et al., 2019). These variables should prompt the surgeon or treating physician to take blood cultures and (if appropriate) perform further diagnostic tests to identify a possible distant infection source.

**Table 9:** A comparison of the failure rates in the literature between patients with bacteraemia and no bacteraemia in early (EA) and late acute (LA) infections after a DAIR procedure.

Literature	DAIR ( n )	Study design	Type of	Failure rate	Failure rate	p value
			infection	bacteraemia	no bacteraemia	
Lora-Tamayo et al. (2013)	345	Retrospective	EA, LA	34/52 (65)	113/276 (41)	<0.001
Lora-Tamayo et al. (2017)	462	Retrospective	EA, LA	63/132 (48)	110/290 (38)	0.02
Löwik et al. (2018)	386	Retrospective	EA	38/73 (52)	110/313 (35)	0.007
Wouthuyzen-Bakker et al. (2019)	340	Retrospective	LA	61/109 (56)	92/230 (40)	0.005
Ludwick et al. (2022)	236	Retrospective	EA, LA	50/103 (49)	44/133 (33)	0.016^*^

^*^ Chi-squared test.

In another multicentre retrospective observational study by Wouthuyzen-Bakker et al. (2020b) comparing late acute ( n=132 ) with early acute infections ( n=132 ), the overall failure rate was higher in late acute PJIs (54 % (late acute) vs. 24 % (early acute); p<0.001 ). Patients with bacteraemia showed an even higher failure rate in both groups (65 % (late acute) vs. 31 % (early acute), p=0.003) . Lora-Tamayo et al. (2017) showed that patients with a late acute infection were more likely to present with bacteraemia, fever, higher leukocyte count, and CRP levels in comparison to early acute infections.

Due to the high failure rate, each of the mentioned studies recommended a two-stage procedure as surgical treatment in patients with bacteraemia or sepsis. During the prosthesis-free interval, microorganisms in the blood system (and at the primary infectious source) can be treated with antibiotics, reducing the risk of continuing hematogenous spread and, hence, a new hematogenous infection at the site of the prosthetic joint. Optimally, a new prosthesis (second stage) should be implanted without the evidence of bacteria in the blood stream. Therefore, if a curative treatment is intended and the patient can tolerate two invasive procedures, a two-stage revision seems to be the current best practice standard of care. It should also be highlighted that in patients with PJI, blood cultures should be taken (especially in patients with acute infections, fever, *S. aureus* infections; Wouthuyzen-Bakker et al., 2019), and a possible primary infectious focus should be identified and treated to prevent subsequent relapse. Indeed, Wouthuyzen-Bakker et al. (2020b) observed a trend towards a higher failure rate in patients with an unidentified source (58.8 %) compared to cases with an identified source (41.2 %) in their multicentre retrospective observational study of 340 late acute infections ( p=0.12 ).

However, if the patient is at high risk or systemically unwell, a local bioburden reduction with a DAIR procedure may be considered. Within the multidisciplinary team further treatment strategies should be discussed. Antibiotic suppressive therapy (in patients at high risk) or a staged procedure (after stabilization of the patient) may be considered afterwards.

#### Recommendation

Based on the current literature, bacteraemia in acute infections is a risk factor for DAIR failure and needs to be considered in the decision-making process.

## 4 Surgical approach

In this section, fundamental surgical principles in undertaking a DAIR procedure with the exchange of mobile components of early and late acute PJIs are described. At first glance, this procedure seems less demanding than other surgical treatment options for PJI, but a meticulous and thorough debridement without removal of vital and functional tissues is of utmost importance to achieve an optimal outcome. For surgeons who are not used to operating on septic cases, it can be very difficult to distinguish between infected, necrotic, and vital tissue. Hence, only surgeons trained in revision arthroplasty should perform these procedures. At the moment, the quality of debridement cannot be measured adequately; however, it is the most important step to achieve infection eradication.

### 4.1 Exchange of mobile parts

Previous studies demonstrated a better outcome of DAIR when mobile parts were exchanged (Table 10) (Lora-Tamayo et al., 2013, 2017; Grammatopoulos et al., 2017a, b; Wouthuyzen-Bakker et al., 2019; Shohat et al., 2020; Svensson et al., 2020; Toh et al., 2021; Zhu et al., 2021; Bernaus et al., 2022). In a large cohort of early and late acute PJIs ( n=1174 , knee and hip), Shohat et al. (2020) observed a higher failure rate in DAIRs without exchange of the mobile components (39 % vs. 30 %, p<0.001 ). In another series of only late acute PJIs of the knee and hip ( n=340 ), the strongest predictor of DAIR success was, indeed, the exchange of mobile components (OR 0.35, 95 % CI: 0.18–0.67, p=0.002 ) (Wouthuyzen-Bakker et al., 2019). Grammatopoulos et al. (2017a) analysed the outcome of only hip PJIs ( n=82 ) and found an almost 5 times increased infection control rate in patients with modular component exchange (OR 4.5, p=0.02 ). Additionally, the chance of 10-year survival improved from 63 % (95 % CI: 44 %–82 %) in DAIR without exchange to 90 % (95 % CI: 80 %–100 %) with exchange in their study ( p=0.01 ) (Grammatopoulos et al., 2017a). A systematic review of 39 articles including 1296 hip PJIs showed a higher mean success rate of 74 % ( n=471 /637, 95 % CI: 70 %–77 %) in patients undergoing modular component exchange compared to 61 % ( n=245 /404, 95 % CI: 56 %–65 %) in patients without exchange (Tsang et al., 2017). In a more recently published large cohort of 575 hip PJI cases of the Swedish Hip Arthroplasty Register, comparable results were observed (Svensson et al., 2020). A significantly lower number of reoperations due to PJI recurrence were seen when mobile components were exchanged at the time of DAIR (HR 0.51, 95 % CI: 0.38–0.68) (Svensson et al., 2020). In knee PJIs, similar outcomes were described in the literature. Zhu et al. (2021) showed in a series of 230 infected total knee arthroplasties that exchanging mobile components was again a predictor of DAIR success in multivariable Cox regression analysis (OR 0.51, 95 % CI: 0.32–0.81, p=0.004 ).

**Table 10:** A comparison of the literature of failure rates following DAIR with or without exchange of the mobile components.

Literature	DAIR ( n )	Study design	Type of	Failure rate	Failure rate	p value
			infection	with exchange	w/o exchange	
Lora-Tamayo et al. (2013)	345	Retrospective	EA, LA	87/212 (41)	42/75 (56)	0.025^*^
Lora-Tamayo et al. (2017)	444	Retrospective	EA, LA	73/211 (35)	98/190 (52)	<0.01
Grammatopoulos et al. (2017a)	82	Retrospective	All PJIs	13/45 (29)	18/37 (49)	0.07
Grammatopoulos et al. (2017b)	122	Retrospective	All PJIs	23/65 (35)	28/57 (49)	0.1
Wouthuyzen-Bakker et al. (2019)	340	Retrospective	LA	64/176 (36)	77/147 (52)	0.004
Shohat et al. (2020)	1174	Retrospective	EA, LA	174/584 (30)	231/590 (39)	<0.001
Svensson et al. (2020)	575	Retrospective	NA	102/364 (28)	93/211 (44)	$<0.0001^{*}$
Toh et al. (2021)	106	Retrospective	EA, LA	16 /60 (27)	16/46 (35)	0.367
Zhu et al. (2021)	230	Retrospective	EA, LA, C	77/186 (41)	29/44 (66)	0.003
Bernaus et al. (2022)	455	Retrospective	EA	35/273 (13)	9/103 (9)	0.297

EA – early acute infection, LA – late acute infection, C – chronic. ^*^ Chi-squared test. NA – not available.

#### Recommendation

Due to the results in the literature, we strongly recommend the exchange of all mobile components during a DAIR procedure. It provides a better visualization and accessibility to the joint (and difficult-to-reach areas) for an adequate and thorough debridement, which is necessary for an optimal bacterial eradication.

### 4.2 Arthroscopic washout vs. DAIR

In 2009, Byren et al. (2009) showed a higher success rate when an open debridement was performed compared to an arthroscopic washout ( n=12 /97, 12 % vs. n=8 /15, 53 %; p<0.0005 ) (Byren et al., 2009). An arthroscopic lavage was associated with a significant risk of failure in their study (HR 4.2, 95 % CI: 1.5–12.5, p=0.008 ). Similar results were reported by Johns et al. (2020) in a cohort of 141 TKAs (success rates of open: n=43 /96, 45 % vs. arthroscopic washout: n=7 /45; 16 %; p<0.01 ). In contrast, findings of a meta-analysis by Kunutsor et al. (2018) suggested higher infection control rates when a DAIR was carried out arthroscopically (72 % vs. 60 %), but results were not statistically significant ( p=0.170 ), and the number of arthroscopic DAIRs was limited ( n=2.712 open DAIRs, n=72 arthroscopic DAIRs). In another pooling analysis of 33 studies consisting of 1266 DAIR cases, the success rate was higher when an open debridement with liner exchange (74 %, 11 studies, n=128 /173, 95 % CI: 67 %–81 %) was done compared with arthroscopic debridement and liner retention (67 %, 4 studies, n=36 /54 cases, 95 % CI: 54 %–79 %), but again the difference was not significant ( p=0.301 ) (Qu et al., 2019).

In the above-mentioned studies, the indications for an arthroscopic or open debridement are not clearly defined. The severity of the infection and patient's comorbidities may vary drastically between cases in these studies. For example, a poor host with multiple comorbidities may prompt the surgeon to perform a less invasive arthroscopic procedure. However, to the best of our knowledge, no well-designed prospective randomized controlled trial with a standardized protocol exists to date that investigated the differences between these two procedures.

In a recently published cohort of 44 PJIs (early and late acute), Bartsch et al. (2023) showed a clear benefit of open debridement compared to arthroscopic lavage. The rate of recurrent infections was significantly higher in the arthroscopic group ( n=10 /13, 77 % vs. n=10 /31, 32 %, p=0.007 ). Their number of included cases was small, but the treatment algorithm was highly standardized (Zimmerli et al., 2004).

#### Recommendation

Although the literature shows ambiguous results, an arthroscopic washout is clearly limited by the inability to perform an adequate debridement and exchange of mobile parts. Hence, to obtain cure, we strongly recommend an open debridement to ensure optimal surgical treatment (adequate and thorough debridement with exchange of mobile parts).

### 4.3 Irrigation solution and volume

Niki et al. (2007) reported that pulsatile lavage with 4 L of sterile saline can remove more than 82 % of bone debris and more than 75 % of cement debris. Due to similar particle sizes, the authors anticipated that bacterial particles may be removed effectively with the same volume of irrigation solution. However, they only analysed the optimal irrigation volume in the setting of a primary TKA and not in septic revision. Hence, possible biofilm formation (immature or mature) was not considered in their study (Niki et al., 2007). In an in vitro study, an irrigation of TKA components could not effectively remove the pre-existing *S. aureus* biofilm formation (Urish et al., 2014). Although a reduction of biofilm mass was observed, a substantial proportion remained on the surface of the TKA material, but irrigation was only performed with 3 L of normal saline solution using high-pressure pulse lavage in their study (Urish et al., 2014). Nevertheless, to date, no clinical data exist on the optimal volume of irrigation when performing a DAIR procedure. In more recently published studies, most institutions used 3–9 L saline for their irrigation during a DAIR procedure (Table 11) (Zhang et al., 2020; Wouthuyzen-Bakker et al., 2020a; Nurmohamed et al., 2021; Tirumala et al., 2021; Toh et al., 2021; Zhu et al., 2021; Chang et al., 2022; Bernaus et al., 2022; Veerman et al., 2022b).

**Table 11:** Used irrigation solutions and volume of irrigation solution per DAIR procedure.

Literature	Study design	PJI	DAIR ( n )	Irrigation solution^*^	Volume per
					procedure
					(L)
Zhang et al. (2020)	Retrospective	EA, LA	24	Saline (pulse lavage)	3L
Wouthuyzen-Bakker et al. (2020a)	Retrospective	EA, LA	455	Irrigation fluid	3–6 L
Nurmohamed et al. (2021)	Retrospective	EA, LA	67	NaCl 0.9 %	6 L
Tirumala et al. (2021)	Retrospective	EA, LA	149	Saline solution + bacitracin	6 L saline
				solution (pulsatile lavage)	2 L bacitracin
Toh et al. (2021)	Retrospective	EA, LA	106	Saline	9 L
Zhu et al. (2021)	Retrospective	EA, LA	230	Wash (pulse lavage)	6 L
Chang et al. (2022)	Retrospective	EA, LA	101	Saline	9 L
Veerman et al. (2022b)	Retrospective	EA	88	Saline (pulse lavage)	6 L
Bernaus et al. (2022)	Retrospective	EA	455	Saline	6–9 L

EA – early acute infection, LA – late acute infection, C – chronic. ^*^ As described in the studies.

Regarding optimal irrigation solution, Siddiqi et al. (2021) performed a review of the literature including all commercially available irrigation solutions for PJI treatment and discussed their advantages and disadvantages. The authors stated that the use of antiseptics (povidone-iodine, chlorhexidine gluconate, acetic acid, hydrogen peroxide, sodium hypochlorite, hypochlorous acid, and preformulated combination solutions) may play a role in PJI treatment. However, they also concluded that there is still a paucity of studies comparing irrigation additives and that treatment protocols between currently available studies are heterogenous, making a direct comparison of different solutions difficult (Siddiqi et al., 2021). In addition, most studies concentrated on PJI prevention rather than PJI treatment. Hence, they were not able to perform a systematic review and meta-analysis or to find the optimal irrigation solution for PJI management based on the current literature (Siddiqi et al., 2021).

It needs to be highlighted that the effect of irrigation on DAIR success alone is difficult to analyse in in vivo studies because of the various other factors influencing the outcome (e.g. chronicity, microorganism, host factors, mechanical debridement).

#### Recommendation

Due to the lack of high-quality data, no evidence-based recommendation on the optimal irrigation solution and volume can be given.

### 4.4 Local antimicrobial treatment and non-traditional antimicrobials

Since penetration of systemic antibiotics can be limited by the presence of necrotic tissue and only few antimicrobial agents are biofilm-active when administered systemically, attention has increased on local use of antibiotics. The application of antimicrobial agents directly into the site of infection provides higher concentrations locally in comparison to systemic antibiotics and may lead to an improved success rate by local bioburden and biofilm reduction.

Vancomycin and gentamicin are commonly used in this setting. In animal and in vitro studies, an anti-biofilm effect was shown when these agents were administered in high concentrations (Okae et al., 2022; Smith et al., 2009). Additionally, gentamicin is a stable antibiotic with activity against most Gram-positive and Gram-negative bacteria and has a synergistic effect with vancomycin against staphylococci (Mulazimoglu et al., 1996). Daptomycin also showed superior activity against biofilm-associated cells in in vitro studies (Smith et al., 2009). However, the choice of the antimicrobial agent(s) also depends on the used carrier, the causing microorganism(s) and its susceptibility (when available), and the host.

Antibiotics can be placed directly into the joint or added to a carrier (Steadman et al., 2023). Mu et al. (2021) demonstrated in their retrospective study of 73 patients with PJI, which occurred within 3 months after primary arthroplasty, a success rate of 88 % when performing a DAIR procedure combined with direct intra-articular antibiotic infusions immediately following DAIR for several days. Chaiyakit et al. (2021) reported similar results (success rate: 87 %) in their retrospective study of 15 acute haematogenous infections treated with DAIR and daily intra-articular antibiotic infusions. In a systematic review by Bruyninckx et al. (2024), three studies including 36 patients treated with DAIR and intra-articular antibiotic infusions were found. The failure rate was 6 % ( n=2 /36). However, the sample size in these studies was small, and there is a potential risk of superinfection and catheter-associated complications when performing this intervention. Due to the limited evidence, the efficacy of a DAIR procedure combined with directly administered antibiotics (powder/fluid) is currently unclear.

In the past decade, antibiotic carriers have become an attractive adjunct for treating PJI due to their high local antimicrobial concentrations (Steadman et al., 2023). While polymethylmethacrylate (PMMA) bone cement beads cannot be recommended due to their specific disadvantages (e.g. antimicrobial resistance, possibility of wear, subsequent surgery for removal), other carriers such as calcium sulfate or hydrogels have recently been used more frequently (Steadman et al., 2023). However, the literature on DAIR involving these carriers loaded with antibiotics is scarce. Calcium sulfate (CS) beads are completely absorbed over a period of several weeks and showed good antimicrobial elution characteristics in the literature. During this absorption period, the antimicrobial content is gradually released, providing high antibiotic concentrations surpassing the minimal inhibitory concentration for common PJI bacteria and superior biofilm reduction/eradication in in vitro studies (Cooper et al., 2016; Sanicola and Albert, 2005; Aiken et al., 2015; Knecht et al., 2018; Dusane et al., 2019; Kallala and Haddad, 2015; McPherson et al., 2022). However, controversial results were reported in in vivo studies. While some authors demonstrated high eradication rates (75 %–88 %) in patients undergoing DAIR involving the application of antibiotic loaded absorbable CS beads (Kallala et al., 2018; Reinisch et al., 2022; Piovan et al., 2022; Sigmund et al., 2024), others were not able to (52 %–55 %) (Flierl et al., 2017; Tarity et al., 2022). Furthermore, some complications such as hypercalcaemia, prolonged wound drainage, and heterotopic ossification can occur (Thwaites et al., 2022; Kallala and Haddad, 2015; Tarar et al., 2021). One small retrospective pilot study of 15 acute PJIs treated with DAIR combined with either hydrogel coating ( n=8 ) or calcium sulfate beads ( n=7 ) as a local antibiotic carrier showed no difference between groups in terms of infection control (87.5 % vs. 100 %, p=0.36 ) (De Meo et al., 2023). However, the sample size was small. Due to the controversial results in the current literature and paucity of reported studies, the effect on outcomes in DAIR procedures using these antibiotic-loaded carriers remains uncertain.

The last emergent option as adjuvant treatment to improve the probability of DAIR success is the local use of non-traditional antimicrobials including bacteriophages, bacteriophage-derived enzymes, and antimicrobial peptides (McCallin et al., 2023; Huang et al., 2022). All these new antimicrobials demonstrated antibiofilm activity in vitro (McCallin et al., 2023). Bacteriophages (or “phages”) are natural viruses with the ability to infect specific pathogenic bacteria. A DAIR procedure involving the use of phages intra- and postoperatively (subsequent local injections under sonography with or without intravenous injections of phages), the so-called “PhagoDAIR”, has been recently described (Ferry et al., 2020, 2024).

Additionally, lysins (enzymes with lytic activity against microorganisms) produced by phages during the infectious process can be applied locally as well (Ferry et al., 2021). Another potential local new antimicrobial treatment option is the use of certain antimicrobial peptides with a broad spectrum and antibiofilm activity (Huang et al., 2022).

However, these non-traditional options are more commonly used in PJI caused by multidrug-resistant microorganisms as a salvage procedure followed by subsequent antimicrobial suppressive therapy. These strategies need to be investigated further.

#### Recommendation

Due to the controversial results in literature and paucity of reported studies, none of the above-mentioned options can be recommended at this stage.

### 4.5 Surgical technique

In DAIR procedures, the previous incision is utilized whenever suitable. To ensure optimal exposure and visualization of the joint, the incision may need to be extended. In case of a suboptimal previous approach, a new incision may be considered. In necrotic or hypertrophic scars, wound margins should be excised. Before arthrotomy, aspiration of the joint to collect synovial fluid for microbiological analysis is performed. Once the joint is open, standardized tissue sampling under strict aseptic precautions should take place. Four to six tissue samples are recommended for microbiological analysis to ensure optimal and reliable results (Dudareva et al., 2018). For each tissue sample, new sterile instruments are advisable to avoid cross-contamination. In patients without sepsis or septic shock, antibiotic treatment can be delayed until all samples have been harvested intraoperatively to increase the identification of the causative microorganism (Singer et al., 2016). For histopathological analysis, three to six tissue samples from areas with high suspicion of infection (e.g. deep soft tissue, (pseudo)capsule, granulation tissue, necrotic tissue, synovia) showed the best performance to provide accurate PJI diagnosis (Sigmund et al., 2023). After tissue sampling, the patient can receive empirical antibiotics intravenously, or (when culture results are available preoperatively) the definitive targeted antibiotics can be started. The next step is the surgical debridement of purulent collections and periarticular tissue. All macroscopically infected, nonviable, and contaminated tissue is meticulously removed, and a synovectomy is performed. The debridement should be thorough and appropriate. Vital tissue and key structures should be preserved (if possible) to avoid unnecessary destabilization of the joint, but any potentially infected and necrotic tissue needs to be excised for infection eradication. The debridement should be systematic and standardized to allow continuity without missing important steps. An adequate debridement is key to DAIR success; hence, it should be performed carefully and precisely. In some institutions, methylene blue is instilled in the joint before arthrotomy (Shaw et al., 2017). This may help to visualize and demarcate infected and inflamed tissue which needs to be excised to ensure precise debridement (Shaw et al., 2017). The mobile parts are then removed using generic or implant-specific explantation devices (polyethylene in knees; acetabular liner and head in hips, in megaprosthesis: modular components). This should be done carefully to avoid damaging the retained prosthesis. Subsequently, the stability and osseointegration of the prosthesis is tested. If the prosthesis is not well integrated and loose, an exchange of the whole prosthesis (one-stage or two-stage revision) is advised. Only in soundly fixed implants should the DAIR procedure be continued.

After optimal exposure following removal of the mobile parts, a further thorough debridement of the previously not visible areas takes place. For dilution of the bacterial bioburden, an extensive irrigation of the tissue, bone, and surface of the retained implant with an abundant amount of 0.9 % sodium chloride is then performed. This may be followed by a wash with an antiseptic solution. In these cases, special attention should be paid to the different soaking times of various available antiseptics to enable the antimicrobial actions of them to work. Additionally, when additive solutions are used, further irrigation with 2 L of saline is needed in most cases.

After extensive lavage, the new mobile components (liner, head) are inserted and impacted. In some rare cases when a new liner and/or head are not available anymore, it is advised to thoroughly clean the removed mobile parts (e.g. brush), soak them in an antiseptic solution, and reimplant them again. However, to the best of our knowledge, no data exist on reused mobile components and their outcome following DAIR procedures. There might be a higher risk of persistence or recurrence of infection which needs to be considered in decision-making and discussed with the patient prior to surgery.

At the end of the procedure, the wound is closed meticulously in layers.

#### Recommendation

A surgeon trained in revision arthroplasty should perform the DAIR procedure.

## 5 Systemic antimicrobial therapy

Choosing the right empirical antimicrobial therapy after surgical debridement, covering the most common causative microorganisms in PJI, is important to enhance DAIR success. A study of Veerman et al. (2022a) demonstrated that an empirical treatment not covering all isolated microorganisms is associated with a higher rate of failure in DAIRs after revision surgery. Therefore, knowing local epidemiology and resistance patterns is crucial to start the appropriate antimicrobial therapy after DAIR. After an induction period of 1 week of intravenous (IV) treatment in which a reduction in bacterial load of mainly planktonic bacteria has been achieved and the isolated microorganisms and susceptibility patterns are known, a switch to oral antibiotic treatment is considered safe. The OVIVA trial, conducted in different types of bone and joint infections, demonstrated non-inferiority of an early switch to oral antibiotics (e.g. within 7 d after surgery) in comparison to longer durations of IV antibiotics (Li et al., 2019). It is important to choose an antibiotic with good oral bioavailability and activity against bacteria embedded in biofilm. Although methodological errors can be noticed in randomized and observational studies regarding oral antibiotic treatment in PJI (Scheper and De Boer, 2022), combination therapy with rifampicin for staphylococci (Table 12; El Helou et al., 2010; Holmberg et al., 2015; Wouthuyzen-Bakker et al., 2019; Eriksson et al., 2023) and the use of fluoroquinolones for Gram negatives is still considered the main stay of antibiotic treatment. El Helou et al. (2010) showed in their study of 86 staphylococcal PJIs that patients treated with DAIR and a rifampicin combination had a lower treatment failure rate in comparison to patients treated without rifampicin (adjusted HR: 0.11, 95 % CI: 0.01–0.84). Holmberg et al. (2015) also demonstrated a 4 times lower failure rate when staphylococcal infections were treated with DAIR and a combination of antibiotics including rifampicin (RR: 4.95 % CI: 2–10). Tai et al. (2022) reported a protective effect when using rifampicin with a lower failure rate in knee PJI after DAIR (HR 0.40, 95 % CI: 0.2–0.8, p=0.008 ), but this was not seen in DAIRs of hip PJIs (HR 1.5, 95 % CI: 0.35–6.2, p=0.597 ). Furthermore, patients with an early rifampicin-resistant staphylococcal PJI had an increased risk of reinfection (HR 1.9, 95 % CI: 1.1–3.6, p=0.04 ) in comparison to rifampicin-sensitive microorganisms when treated with DAIR in a retrospective single-centre study by Eriksson et al. (2023). In a meta-analysis by Kruse et al. (2022), rifampicin showed a significant reduction in failure rates when performing DAIR selectively in 1103 patients (OR 0.47, 95 % CI: 0.3–0.8, p=0.005 ). However, in another meta-analysis on DAIR and rifampicin use, the pooled risk ratio for rifampicin effectiveness was only 1.10 (95 % CI: 1.00–1.22) (Scheper et al., 2021). For staphylococci, a fluoroquinolone is considered the most appropriate backbone of rifampicin (Beldman et al., 2021). Interactions of rifampicin with other antibiotics should be taken into consideration when choosing a co-drug for rifampicin (Tornero et al., 2016), although these interactions are not always clinically relevant when the co-drug is dosed appropriately (Beldman et al., 2021). The use of rifampicin for other Gram-positive bacteria, like *Cutibacterium acnes* or *Streptococcus* species, can be considered, but there is limited evidence to support its routine practice (Lora-Tamayo et al., 2017; Kusejko et al., 2021).

**Table 12:** A comparison of the literature between antimicrobial regimens with and without rifampicin after a DAIR procedure.

Literature	DAIR ( n )	Study design	Microorganism	Antimicrobial regimen	FR with	FR without	p value
					rifampicin	( n ; %)	
					( n ; %)		
El Helou et al. (2010)	101	Retrospective	Staphylococci	Rifampicin/levofloxacin ( n=14 ) vs.	1/14 (7)	21/56 (38)	0.029
				no rifampicin regimen ( n=56 )			
Holmberg et al. (2015)	86	Retrospective	Staphylococci	Combination + rifampicin vs. no rifampicin	13/69 (19)	10/17 (59)	0.01
Wouthuyzen-Bakker et al. (2019)	165	Retrospective	Staphylococci	Combination + rifampicin vs. no rifampicin	66/134 (49)	21/31 (68)	0.06
Eriksson et al. (2023)	81	Retrospective	Staphylococci	Rifampicin-sensitive vs. rifampicin-resistant	31/61 (51)	16/20 (80)	0.002

FR – failure rate.

For Gram-negative bacteria (GNB), the use of fluoroquinolones can be recommended. In a prospective study of 47 PJI cases caused by GNB, the treatment with fluoroquinolones was associated with a better outcome following DAIR (OR 9.09, 95 % CI: 1.96–50, p=0.005 ) (Martínez-Pastor et al., 2009). In another retrospective multicentre study of 174 acute GNB PJI cases, the use of ciprofloxacin was an independent factor for DAIR success (HR 2.56, 95 % CI: 1.14–5.77, p=0.02 ) (Rodríguez-Pardo et al., 2014). The use of fluoroquinolones for GNB is also supported by in vitro data in which fluoroquinolones demonstrated the best activity against in biofilm-embedded bacteria (Abdi-Ali et al., 2006; Yassien et al., 1995; Di Bonaventura et al., 2004). However, comparative studies with, for example cotrimoxazole, have not been performed. The total duration of antibiotic treatment after DAIR remains a matter of debate. Some studies demonstrated an excellent outcome after 6–8 weeks of antibiotic treatment (Lora-Tamayo et al., 2016; Chaussade et al., 2017), but often these studies were performed in a selected group of patients. The landmark DATIPO trial published in 2021 demonstrated an inferior outcome of 6 weeks of antibiotic treatment in comparison to 12 weeks (Bernard et al., 2021). For this reason, a total antibiotic duration of 12 weeks after DAIR is still recommended. Future studies are needed to identify which categories of patients are eligible for a shorter duration and which patients are candidates for suppressive antibiotic treatment.

**Table 13:**
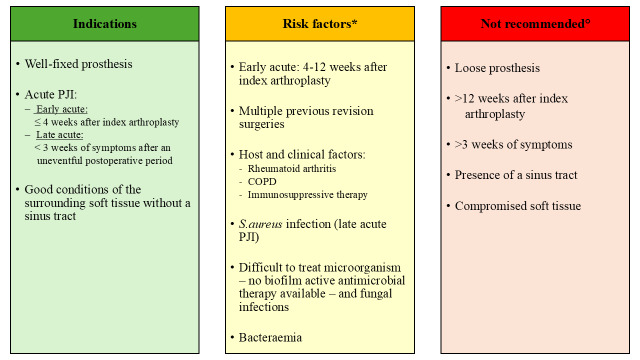
Indications, risk factors to consider in the decision-making process, and contraindications for a DAIR (debridement, antimicrobial therapy, and implant retention) procedure in periprosthetic hip and knee infections when cure is intended based on the current literature.

^*^ These host and clinical factors can be associated with a higher risk of failure. ° If cure is intended, in patients fulfilling these factors, an exchange of the whole implant should be considered.

### Recommendation

A total antibiotic duration of 12 weeks after DAIR including an induction period of 1 week of intravenous (IV) treatment can be recommended. For staphylococci, a combination of a fluoroquinolone with rifampicin is recommended and for Gram-negative fluoroquinolones.

## 6 Conclusion

DAIR is an efficient treatment modality with good eradication rates in carefully selected patients (Clauss et al., 2020). It is an attractive option for surgeons as it is technically less demanding and may prevent unnecessary removal of a well-fixed prosthesis. Patients can return to activity more quickly, and the overall economic cost is lower. However, not all patients benefit from a DAIR procedure. It has been shown that the outcome depends on various factors (e.g. type of infection, bacteria, host and clinical factors, soft tissue envelope, causing pathogen, surgical technique) and that patient selection is key for DAIR success. Hence, we tried to elucidate the most relevant host, clinical, and surgical factors for DAIR success as well as DAIR failure in this guideline. Based on the current literature, we tried to define the indications and contraindications for a DAIR procedure in acute PJIs as well as risk factors for DAIR failure (as shown in Table 13).

However, it needs to be highlighted that results are often inconsistent, and the impact of various factors on DAIR outcomes is still unclear in the available literature. Additionally, tremendous variability between studies regarding DAIR indication (e.g. type of infection, definition of acute infection), study population, confounding factors (e.g. autoimmune disorders, cancer, age, sex, underlying diseases, medications), definition of treatment success and failure, length of follow-up periods, spectrum of causing microorganisms, surgical techniques, antimicrobial therapy, and number of performed DAIRs was shown. Hence, the reported populations were very heterogenous. Furthermore, most studies were retrospectively designed, were underpowered due to the small number of included cases, and gave limited insights into their methodology. All this limits the strength of evidence and complicates interpretation and comparison between studies. Well-designed prospective enrolled studies and randomized trials with standardized protocols are needed to optimize indications and surgical techniques in the future. Machine learning with the ability to learn from data input may also help us to gain more knowledge about the important risk factors for DAIR failure and predictors of DAIR success in the future. However, we tried our best to establish a list of indications and contraindications to achieve the optimal outcome based on the currently available literature (Table 13).

**Table 14:** Important steps during a DAIR (debridement, antibiotics, and implant retention) procedure.

Surgical technique
1. open arthrotomy
2. standardized deep tissue sampling (1 aspiration, 4–6 tissue samples)
3. adequate and standardized surgical debridement
4. removal of the mobile parts
5. stability testing of the prosthesis (well fixed)
6. second thorough debridement
7. irrigation
8. insertion of new mobile parts

Nevertheless, there may also be patients who do not fulfil these criteria but could still be considered for a DAIR procedure. Patients with a chronic infection but at high risk and/or for whom alternative surgical treatment options are unacceptable may also undergo a DAIR procedure. The high failure rate in these patients ( >50 %) needs to be discussed in a multidisciplinary team and with the patient. It is then seen as bioburden reduction rather than infection eradication (no curative intention). However, the benefits must be weighted against the high failure rate and possible adverse effects of this surgical intervention to find the optimal treatment strategy for these patients.

We also want to emphasize that the final decision of the PJI management is up to the treating multidisciplinary team (orthopaedic surgeon, ID physician, microbiologist, radiologist, plastic surgeon) and the affected patient and should be made on a case-by-case basis. Additionally, the implementation of our recommendations may not be possible in all hospitals or institutions. In such cases, a referral to a specialized infection centre might be advisable.

We hope that this guideline may help reduce the reinfection rates as well as the physical, psychological, and economic burden associated with PJI. We believe that a reasonable outcome can be achieved with careful patient selection (Table 13), a dedicated multidisciplinary team, and an appropriate surgical technique (Table 14).

## Data Availability

All data generated or analyzed in this position paper are included in the published article.
